# Design and fabrication of 1D nanomaterials for electromagnetic wave absorption

**DOI:** 10.1093/nsr/nwae420

**Published:** 2024-11-22

**Authors:** Hongdu Jin, Min Liu, Lei Wang, Wenbin You, Ke Pei, Han-Wen Cheng, Renchao Che

**Affiliations:** Laboratory of Advanced Materials, Shanghai Key Lab of Molecular Catalysis and Innovative Materials, Department of Materials Science, Academy for Engineering & Technology, Fudan University, Shanghai 200438, China; Laboratory of Advanced Materials, Shanghai Key Lab of Molecular Catalysis and Innovative Materials, Department of Materials Science, Academy for Engineering & Technology, Fudan University, Shanghai 200438, China; Laboratory of Advanced Materials, Shanghai Key Lab of Molecular Catalysis and Innovative Materials, Department of Materials Science, Academy for Engineering & Technology, Fudan University, Shanghai 200438, China; Laboratory of Advanced Materials, Shanghai Key Lab of Molecular Catalysis and Innovative Materials, Department of Materials Science, Academy for Engineering & Technology, Fudan University, Shanghai 200438, China; Laboratory of Advanced Materials, Shanghai Key Lab of Molecular Catalysis and Innovative Materials, Department of Materials Science, Academy for Engineering & Technology, Fudan University, Shanghai 200438, China; Laboratory of Advanced Materials, Shanghai Key Lab of Molecular Catalysis and Innovative Materials, Department of Materials Science, Academy for Engineering & Technology, Fudan University, Shanghai 200438, China; Laboratory of Advanced Materials, Shanghai Key Lab of Molecular Catalysis and Innovative Materials, Department of Materials Science, Academy for Engineering & Technology, Fudan University, Shanghai 200438, China; College of Physics, Donghua University, Shanghai 201620, China; School of Materials Science & Engineering, Tongji University, Shanghai 201804, China

**Keywords:** electromagnetic wave absorption, one-dimensional nanomaterials, absorption mechanism, fabrication methods

## Abstract

The design and fabrication of high-performance electromagnetic wave (EMW) absorbing materials are essential in developing electronic communication technology for defense and civilian applications. These materials function by interacting with EMWs, creating various effects such as polarization relaxation, magnetic resonance, and magnetic hysteresis in order to absorb EMWs. Significant progress has been made to improve the dimensional performance of such materials, emphasizing the ‘thin, light, broad, and strong’ functional specifications. One-dimensional (1D) nanostructures are characterized by high surface area, low density, and unique electromagnetic properties, providing promising solutions to address some of the challenges in facilitating multiple reflections and wideband resonances, which are crucial for effective EMW attenuation. This paper provides an overview of recent advances in exploring 1D structures for enhancing EMW absorption and their controllability. The design and fabrication of nanofibers, nanowires, and other 1D nanostructures are highlighted. The advantages of 1D nanomaterials in EMW absorption are also described. Challenges and future directions are discussed, focusing on developing new design concepts and fabrication methods for achieving high-performance and lightweight EMW absorbers and enhancing fundamental understanding of EMW absorption mechanisms.

## Teaser

One-dimensional nanostructures enable thinner, lighter, broader, and stronger electromagnetic wave absorption. The mechanistic understanding, as highlighted in this review, will advance their design and fabrication in order to achieve unprecedented higher performance.

## INTRODUCTION

**Figure 1.**
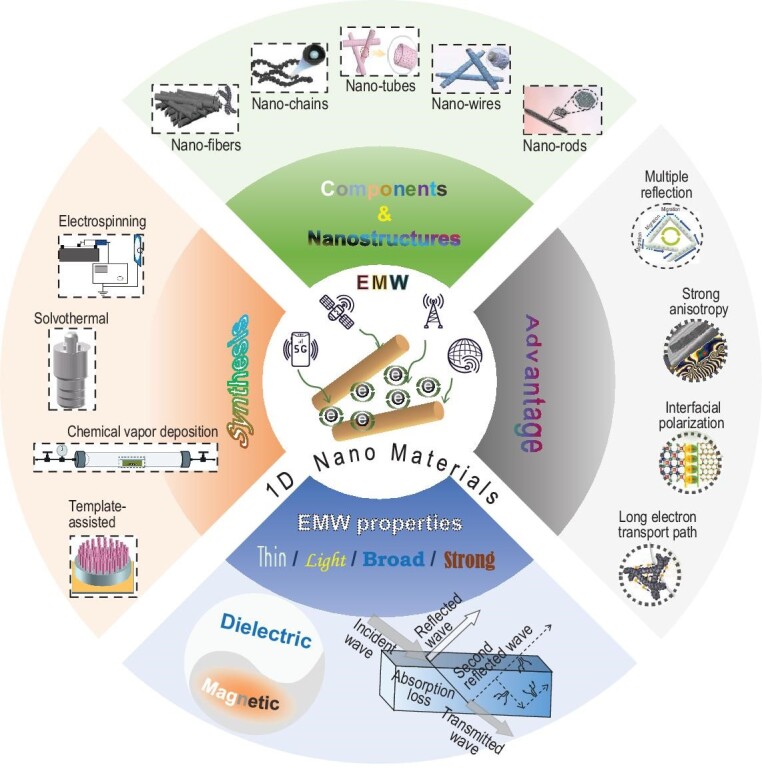
Illustration of one-dimensional (1D) nanomaterials with different compositions and microstructures for EMW absorption.

With the rapid development of modern electronic communication technology, especially the widespread application of 5G mobile technology, wireless networks have been deeply integrated into various aspects of human life [1–3]. However, the increasing use of wireless communication devices has resulted in growing EMW radiation pollution, which is emerging as a significant concern and poses potential risks to human health [4]. Therefore, there is an urgent need for developing ‘wideband (wide), lightweight (light), thin, and strong-absorption (strong)’ EMW absorbing materials [5–7] (Fig. 1).$\rule{16.46pc}{0.5pt}$

$\rule{16pc}{0.5pt}$ EMW is a well-known form of energy system in which electric and magnetic fields propagate perpendicular to each other. The attenuation of EMWs is primarily caused by dielectric loss and magnetic loss [8]. According to the transmission line theory [9,10], the absorption performance of materials entails the understanding of the impedance matching conditions (|Z_in_/Z_0_|) and attenuation characteristics (*α*) of materials to EMWs [11]. Under certain frequency conditions, the impedance matching and attenuation constants are calculated as follows [12]:


(1) \begin{eqnarray*}\left| {\frac{{{{Z}_{{\mathrm{in}}}}}}{{{{Z}_0}}}} \right| = {{\left( {\frac{{{{\mu }_r}}}{{{{\varepsilon }_r}}}} \right)}^{\frac{1}{2}}}\tanh \left\{ {j\left( {\frac{{2\pi fd}}{c}} \right){{{\left( {{{\mu }_r}{{\varepsilon }_r}} \right)}}^{\frac{1}{2}}}} \right\},\end{eqnarray*}

(2) \begin{eqnarray*}
\alpha = \frac{{\sqrt 2 \pi f}}{c}\sqrt {\left( {\mu ^{\prime\prime}\varepsilon ^{\prime\prime} - \mu ^{\prime}\varepsilon ^{\prime}} \right) + \sqrt {{{{\left( {\mu ^{\prime\prime}\varepsilon ^{\prime\prime} - \mu ^{\prime}\varepsilon ^{\prime}} \right)}}^2} + {{{\left( {\mu ^{\prime}\varepsilon ^{\prime\prime} + \mu ^{\prime\prime}\varepsilon ^{\prime}} \right)}}^2}}},
\end{eqnarray*}

where *c* is the speed of light, *μ′* and *μ″* are the real and imaginary parts of the permeability, respectively. *ε*′ and *ε*″ are the real and imaginary parts of the permittivity, respectively. The impedance matching conditions determine whether the incoming EMWs can enter the absorber. The ideal impedance matching condition refers to the situation where incoming waves can fully enter the absorber with zero reflection [13]. According to Equation (1), when the thickness (*d*) of the absorber and the frequency (*f*) of the EMW are fixed, the impedance matching value (|Z_in_/Z_0_|) of the absorber is only related to the complex permittivity (*ε*_r_) and complex permeability (*μ*_r_) of the material [14]. Therefore, adjusting the balance between electromagnetic properties is a key step in achieving impedance matching. The attenuation capability determines whether EMWs inside the material can be dissipated [15]. Excellent EMW absorption performance can only be achieved when both prerequisites are met. Fulfilling these two prerequisites requires a comprehensive consideration of the electromagnetic parameters of the materials in the design to achieve minimized reflection losses (RL_min_) [16]:


(3) \begin{eqnarray*}{\mathrm{R}}{{{\mathrm{L}}}_{{\mathrm{min}}}} = 20{\rm log}\left| {\frac{{{{Z}_{{\mathrm{in}}}} - 1}}{{{{Z}_{{\mathrm{in}}}} + 1}}} \right|.\end{eqnarray*}

In addition to the intrinsic properties of materials, such as magnetic and dielectric characteristics, the morphological structure also significantly influences their EMW absorption performance. Parameters such as the distribution of heterogeneous interfaces, conductivity, magnetic permeability, and EMW transmission paths are intricately linked to the morphological structure [17]. Constructing specific ordered structures on the surface and within the material can improve free-space impedance matching, allowing EMWs to penetrate more effectively. Recently, researchers have synthesized a myriad of well-designed nanostructures, including nanoparticles (NPs) [5], nanoarrays [18], nanofilaments [19], and nanosheets [20]. Among these structures, 1D nanomaterials such as nanofibers (NFs) [16], nanowires (NWs) [21], nanochains (NCs) [22], nanotubes (NTs) [23], and nanorods (NRs) [24] stand out due to several advantages (Fig. 1). First, these 1D structures can overcome the limitations of isotropic materials in terms of electromagnetic parameters. Dipole interactions and magnetic anisotropy are modulated by adjusting the composition, particle size, diameter, and aspect ratio, making it easier to surpass the Snoek limit ($( {{{\mu }_i} - 1} ){{f}_0} = \gamma {{M}_S}/3\pi $) [25]. Theoretical analysis has confirmed that the construction of 1D magnetic nanostructures is a feasible solution to tailor the impedance and dissipation ability in low-frequency bands, thereby improving the microwave absorption efficiency of materials. Second, 1D structures have low percolation thresholds and high aspect ratios, and provide direct paths for charge transport, facilitating the electron transfer paths and improving conduction losses [26]. Third, the high surface area and strong interface polarization effects of 1D structures also enhance dielectric loss [27]. Finally, 1D nanomaterials serve as the building blocks contributing to the formation of three-dimensional (3D) conductive network structures. The interwoven conductive networks promote multiple scattering and reflection of EMWs. The repeated scattering of EMWs among 1D nanomaterials leads to cross-polarization, thereby dissipating EMWs [28].

In recent years, 1D nanomaterials have garnered widespread attention due to their significant potential in EMW absorption. Some of the important aspects of 1D nanomaterials for microwave absorption have been highlighted by outstanding reviews on certain materials such as carbon-based [10,29,30], targeted preparation methods such as electrospinning [31], and specific structures such as porous/core-shell/hollow [32]. In this context, the present review aims to systematically discuss the latest developments and research trends of 1D nanomaterials with different morphologies, such as nanofibers, nanowires, nanochains, nanotubes, and nanorods, from the following aspects. First, the review covers various advanced methods that have stood the test of time (to date) to fabricate 1D nanostructures. These methods not only enable precise control of the morphology and dimensions, but also allow further optimization of their electromagnetic properties through element doping and composite formation. Second, a detailed analysis of the microstructural characteristics of different 1D nanomaterials and their effects on EMW absorption performance is provided. The distinctive advantages of high aspect ratio, significant anisotropy, and excellent electromagnetic properties in enhancing dielectric and magnetic losses are emphasized. Furthermore, the potential of 1D nanomaterials to meet the requirements of ‘wide, light, thin, and strong’ wave-absorbing performance is discussed. Finally, the challenges and prospects of 1D nanomaterials for future applications are discussed. By thoroughly analyzing current research findings and existing problems, the review discusses some valuable application insights, which are of great significance for the development of a new generation of high-efficiency EMW absorbing nanomaterials.

## FRONTIER FABRICATION METHODS OF 1D EMW ABSORBERS

The morphology and structure of EMW absorbers critically influence their absorption performance. Key parameters such as heterogeneous interface distribution, conductivity, magnetic permeability, and EMW transmission paths are closely linked to the material's morphology. Numerous well-designed nanostructures have been successfully fabricated and reported to date [33,34]. One-dimensional structures have garnered significant attention due to their unique advantages, including high surface area, strong anisotropic shape, and excellent electron transport properties. These 1D nanostructures significantly enhance EMW absorption by increasing the absorption cross-section and leveraging nanoscale quantum and interfacial polarization effects. Additionally, these effects introduce new pathways for absorption enhancement. Advanced methods for preparing 1D EMW absorbers currently include electrospinning, solvothermal/hydrothermal methods, chemical vapor deposition, template-assisted techniques, magnetic-field–induced self-assembly, chemical methods, 3D printing technology, and so on. The application of these techniques enables researchers to precisely control the morphology and dimensions of nanostructures as well as their performance. The following numerous examples demonstrate that these innovations continue to support the development of high-performance EMW absorbing materials.

### Electrospinning method

The electrospinning method is recognized as one of the most innovative and promising techniques for fabricating 1D materials. By applying high voltage to a polymer solution, induced electrostatic forces stretch the polymer into fine fibers [35–38]. Its inherent simplicity, scalability, and capability to incorporate functional additives or composites make it widely applicable in the field of EMW absorption [39]. During electrospinning, a high-voltage electrostatic field electrically charges the precursor solution. As the voltage increases, a characteristic ‘Taylor cone’ is formed. The polymer solution overcomes the surface tension and creates a high-velocity jet that is deposited as nanofibers onto the collector plate. Precise control of fiber diameters and aspect ratios is achieved by adjusting parameters such as spinning voltage, fluid properties, temperature, and spinning distance [40].

**Figure 2.**
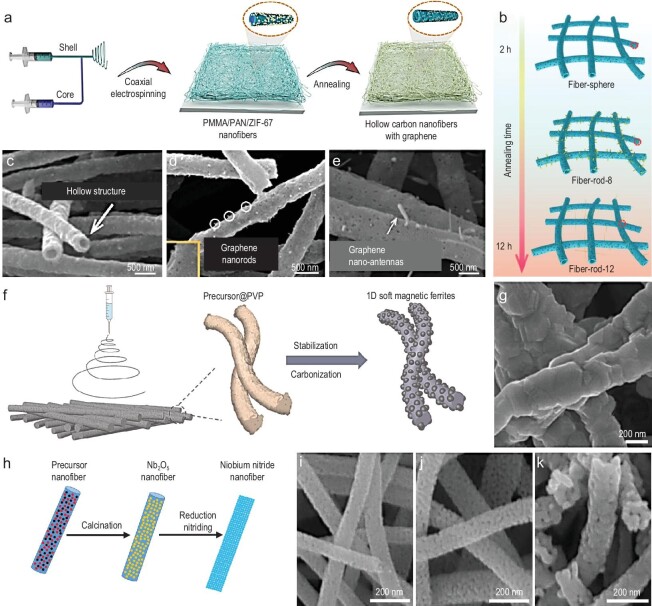
(a and b) Schematic illustration of the preparation process and microstructure evolution of hollow carbon nanofibers with graphene with increasing annealing time. (c) SEM image of the hollow structure of carbon nanofibers with graphene spheres. (d) SEM image of Fiber-Rod-8 samples which were obtained by the annealing treatment of precursors for 8 h. (e) SEM image of graphene nano-antennas samples. Adapted with permission from ref. [44]. Copyright 2023, American Chemical Society. (f) Schematic illustration of the fabrication process of 1D magnetic ferrites. (g) SEM image of MnZn magnetic ferrites. Adapted with permission from ref. [28]. Copyright 2023, Elsevier. (h) Schematic illustration of the preparation process of niobium nitride porous nanofibers. (i–k) SEM images of the niobium nitride nanofibers reduced nitrate at 700, 800 and 900°C. Adapted with permission from ref. [46]. Copyright 2022, Elsevier.

The electrospinning method is known for its simplicity, rapid processing, and versatility in spinning various types of 1D nanomaterials. It enables the fabrication of diverse nanomaterials including ferrites [41,42], magnetic alloys [43], 1D carbon-based materials [44], and multicomponent composites [45]. For example, the electrospinning calcination method allows metal salts uniformly distributed within the fibers to be converted into metal oxides through high-temperature calcination, resulting in the synthesis of NiZn ferrite nanofibers [42]. By optimizing the nanofiber diameter, coercivity, saturation magnetization, and electromagnetic properties, the nanofibers achieve a reflection loss < −10 dB across the entire X-band frequency range. The significant gaps between NiZn ferrite nanofibers can effectively introduce an air phase, thereby adjusting the dielectric constant and reducing the density. Further structural optimizations, such as hollow and core-shell configurations, provide additional benefits by reducing weight and leveraging structural advantages. Fan *et al*. [44] used coaxial electrospinning technology to fabricate 1D hollow carbon nanofibers combined with graphene nanorods (Fig. 2a). The morphology of the composite can be controlled by the annealing process (Fig. 2b–e). These nanorods function as nano-antennas, effectively directing EMWs into the materials to improve impedance matching conditions. Consequently, at a thickness of 4.6 mm, the composite achieves an RL_min_ of −57.1 dB and an effective absorption bandwidth (EAB) that covers almost the range of 2.4∼8 GHz. Compositing magnetic materials (such as alloys and ferrites) with carbon-based materials can enhance magnetic permeability and optimize impedance matching. For instance, multi-interfacial 1D magnetic ferrite@carbon fibers constructed through coaxial electrospinning demonstrated improved EMW performance [28] (Fig. 2f). Due to the strong polarization and magnetic coupling interaction network of 1D anisotropy, MnZn ferrite@carbon fibers attain impressive RL_min_ of −47.4 dB and EAB of 9.2 GHz (8.24∼17.52 GHz). Electrospun nanotubes form interconnected conductive networks that facilitate the increase of electron transfer pathways (Fig. 2g). Beyond carbon-based materials, other types of electrospun nanofibers, such as porous niobium nitride nanofibers, also perform well. The porous nanofibers synthesized through electrospinning formed conductive networks within the fibers, endowing them with outstanding EMW absorption capabilities [46] (Fig. 2h–k). With a matching layer thickness of only 2.04 mm, the porous niobium nitride nanofibers exhibited an optimal reflection loss of −49.5 dB at 8.9 GHz.

Through the electrospinning technique, various types of 1D nanomaterials can be prepared with uniform composition distribution and unique physicochemical properties. These electrospun nanomaterials can form interconnected conductive networks that increase electron transfer pathways and promote EMW absorption. While electrospinning enables the fabrication of elongated nanofibers, its high cost and limited control over fine structures render it unsuitable for large-scale production. Still, process optimization, precise control of structural parameters, and exploration of new material choices and design strategies can be employed to improve the physical and chemical properties of nanofibers for EMW absorption and other applications.

### Solvothermal/hydrothermal method

The solvothermal/hydrothermal method involves the preparation of materials through chemical reactions in an aqueous environment under controlled conditions [47]. Under high temperature and pressure conditions, the precursor is influenced by the solvent effect of water and thermodynamic effects to form various unique structures, such as nanotubes, nanorods, and nanowires. The morphology, size, and structure of the 1D materials can be precisely controlled by adjusting temperature and pressure, as well as reaction time.

**Figure 3.**
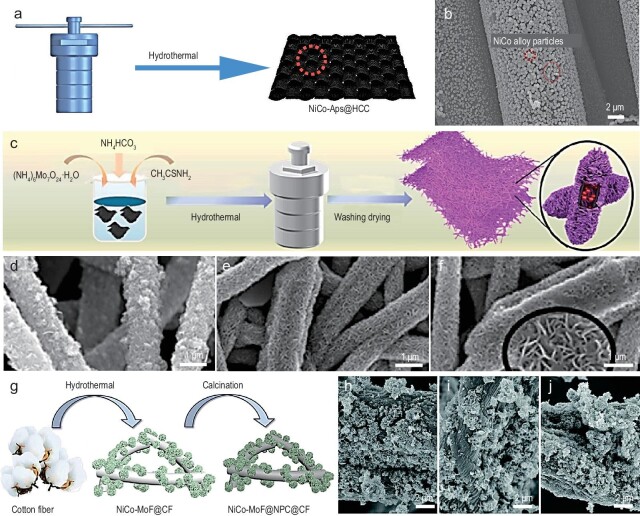
(a) Schematic illustration of the preparation process of NiCo alloy particles @ hydrophilic carbon cloth (NiCo-APs@HCC). (b) SEM images of NiCo-APs@HCC composites. Adapted with permission from ref. [49]. Copyright 2022, Elsevier. (c) Schematic illustration of the formation of the carbon fiber/Mil88A/MoS_2_ (CMM) samples. (d) SEM image of carbon fibers. (e and f) SEM images of CMM. Adapted with permission from ref. [52]. Copyright 2023, Elsevier. (g) Synthesis process of NiCo@nanoporous carbon@carbon fiber nanocomposites (NiCo@NPC@CF). (h–j) SEM images of NiCo@nanoporous carbon@carbon fiber with annealing temperatures increasing (600°C, 700°C, and 800°C). Adapted with permission from ref. [16]. Copyright 2022, Royal Society of Chemistry.

Typically, solvothermal/hydrothermal methods are primarily employed to synthesize 1D EMW absorbing materials through two main approaches. The first approach involves direct preparation using the above methods. For example, flower-like composite aerogels can be prepared via a simple hydrothermal method [48]. In this process, the 1D Fe_3_O_4_@SiO_2_@MnO_2_ nanochains induce the curling of graphene oxide (GO), encapsulating it within the curled structure. The aerogels exhibit excellent EMW absorption performance due to their well-designed structural interfaces. Liu *et al*. [49] designed NiCo alloy particles@hydrophilic carbon cloth composites with a unique corncob-like structure using an *in-situ* hydrothermal method (Fig. 3a). By adjusting the Ni/Co ratio, the crystal structure can be tuned. In addition, different interfaces and defects that induced dipole and interface polarization can be introduced, optimizing the EMW absorption performance. The EAB nearly covers the entire Ku band (Fig. 3b). In a previous report, Fe nanowires composed of interconnected bead-like NPs were fabricated using a magnetic-assisted hydrothermal method [50]. The diameter and length of these nanowires are fine-tuned by optimizing the precursor molar ratios, reaction time, and temperature, resulting in impressive absorption performance in the low-frequency GHz range, with an RL_min_ of −27.28 dB at 3.68 GHz. Traditional solvothermal methods have also been used to synthesize magnetic chain-like ferrites such as Fe_3_O_4_, NiFe_2_O_4_, and CoFe_2_O_4_ [51]. These chain-like structures control electromagnetic parameters and significantly enhance absorption performance, achieving an optimal EAB of 6.4 GHz with a thickness of 1.8 mm.

Another preparation method involves the synthesis of 1D precursors, such as metal-organic frameworks (MOFs), through hydrothermal reactions and calcination to obtain materials that inherit the morphology of the precursors. The magnetic flower-shaped nanorods (CMM), derived from MOF (Mil-88A) are synthesized via a straightforward hydrothermal route followed by annealing [52] (Fig. 3c–f). Due to their unique 1D flower-like wrinkled structure, these nanorods exhibit remarkable EMW absorption abilities. Specifically, the CMM-900 (carbonization temperature ∼ 900°C,) achieves a reflection loss of up to −70 dB with a thickness of merely 2.5 mm, surpassing the performance of most absorptive materials. Recently, Sun's group employed a solvothermal method to prepare Co_3_O_4_ nanotubes, which were further used as self-sacrificial precursors after *in-situ* polymerization to coat them with a phenolic resin layer [53]. Subsequent high-temperature carbonization resulted in the fabrication of a yolk-shell Co@C nanotube. Leveraging the 1D stacked structure to reduce electron migration barriers, they effectively achieved a strong absorption at −51.4 dB with low powder filling rates. A similar method has also been used to elaborate the microstructure and interface of composites, resulting in the fabrication of CoTe_2_ nanorods combined with MoS_2_ nanosheets to form nanocomposites [54]. These composites have remarkable EMW absorption capability, primarily due to their 1D network structure. The latter allows multiple reflect and scatter, resulting in significant attenuation.

Achieving broadband absorption is a well-known challenge. The combination of multidimensional materials can facilitate the formation of numerous interfaces, and strong interfacial interactions lead to polarization. Heterostructural composites can also enhance impedance matching to achieve broadband absorption. Jin *et al*. [16] synthesized a 1D composite material by uniformly growing flower-like NiCo-MOF particles on cotton fibers using a hydrothermal method, followed by annealing in an N_2_ atmosphere (Fig. 3g). This composite exhibits a broad EAB (10.0 GHz) across the 8∼18 GHz frequency range through hierarchical structural construction (Fig. 3h–j).

Although the solvothermal/hydrothermal method is widely used for synthesizing 1D nanomaterials, it has drawbacks. For example, long reaction times, high temperature and pressure requirements, and the potential degradation of organic compounds lead to reduced product purity and crystallinity. To address these issues, optimizing reaction conditions and using appropriate catalysts can limit reaction time and energy consumption while improving product purity and crystallinity.

### Chemical vapor deposition method

**Figure 4.**
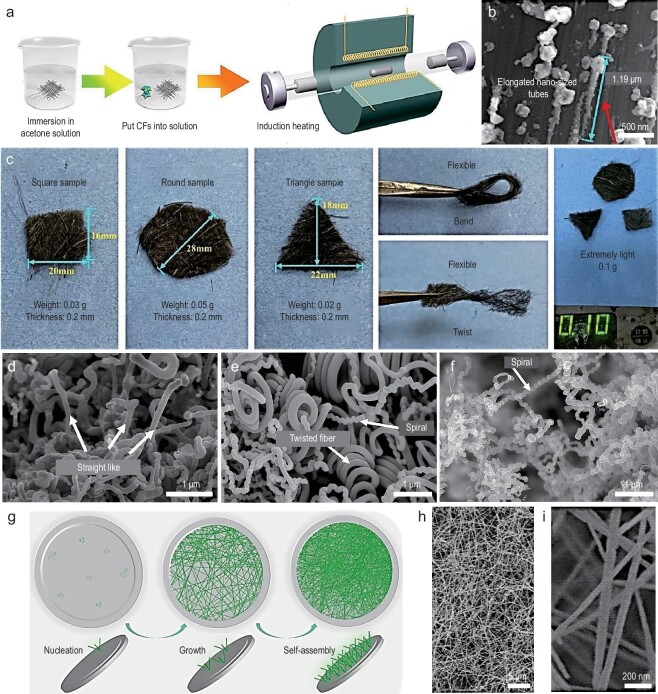
(a) Schematic illustration of the preparation process of CNT/CFs composites. (b) SEM image of CNTs. (c) Images of sample preparation of CNTs/CF composites. Adapted with permission from ref. [55]. Copyright 2021, Elsevier. (d–f) SEM images of CNT catalyzed by mill scale with different milling time (4 h, 20 h, and 40 h). Adapted with permission from ref. [57]. Copyright 2022, Springer Nature. (g) Illustration of the preparation of silicon carbide nanowire aerogels (SNWA). (h and i) SEM images of SNWA samples. Adapted with permission from ref. [58]. Copyright 2024, Elsevier.

Chemical Vapor Deposition (CVD) method is a common technique for the preparation of 1D nanomaterials. Carbon-hydrogen compound gases serve as carbon sources, while elements such as cobalt, nickel, and iron serve as catalysts. Under certain temperature, pressure, and atmospheric conditions, compound elements evaporate and deposit on the substrate surface to form the desired product. CVD is employed in the growth of materials such as carbon nanotubes, graphene, and metal films, offering high efficiency, controllability, low cost, and scalability. Zhan and co-workers used a CVD-based induction-heating method to grow CNTs (carbon nanotubes) directly onto CFs (carbon fibers) [55] (Fig. 4a). By controlling the length of the CNTs, they obtained a 1D composite with a large specific surface area and multi-level heterogeneous interfaces (Fig. 4b–c). The composite shows optimal performance with a filling rate of 1% and a thickness of 3 mm, characterized by strong absorption, thin thickness, and wide bandwidth (RL_min_ = −44.4 dB, and EAB = 7.4 GHz). This research not only provides new insights for the development of efficient EMW absorption materials, but also offers inspiration for exploring multi-level interface effects and optimizing heterogeneous interfaces in composite design.

In the process of synthesizing CNTs and CF via the CVD method, many aspects need to be noted. Not only the process parameters such as carbon sources, catalysts, and reaction temperature need to be well set, but also the type of reactor should be chosen judiciously to create the airflow characteristics required for fiber spinning. Varying airflow distributions within the reactor are reported to have distinct effects on the generation and trajectory of CNTs, thereby influencing their ultimate quality. As a case in point, Liu's group [56] synthesized different CNT- and La-doped CoFe_2_O_4_ composites using the CVD technique. By adjusting the deposition time, they controlled the length, density, and content of CNTs in the composites. The improved impedance matching of the micro-heterogeneous structures, along with the synergistic effects of increased dielectric and magnetic losses, resulted in an RL_min_ of −35.3 dB. Recently, 1D Fe_3_C/C composites with diverse morphologies (Fig. 4d–f) were obtained by the CVD technique [57]. By varying the grinding time of the catalyst (4∼40 hours), Fe_3_C/C composite formed twisted, helical, and spring-like structures with multi-level heterogeneous interfaces. The spiral-shaped composite exhibited optimal EMW absorption performance when the sample was ground for 20 hours. In another work, Wang's group fabricated silicon carbide nanowire aerogels (SNWAs) using a straightforward CVD process, subsequently modifying the SiO_2_ layer on SiC nanowires through controlled oxidation [58] (Fig. 4g–i). By optimizing the oxide layer, they enhanced impedance matching, amplified defect-induced dipole polarization, and maintained high interface polarization loss. These techniques achieved strong absorption with an RL_min_ of −57.2 dB and a wide EAB spanning 9.4 GHz (8.6∼18 GHz).

Despite the numerous advantages of the CVD method in synthesizing 1D nanomaterials, it still faces challenges. First, high temperature and high vacuum demand high equipment stability and cost. Second, it is prone to toxic and flammable by-products. To address these issues, researchers are currently exploring environmentally friendly CVD techniques, such as reducing temperature and pressure, optimizing reaction parameters, developing safer precursors and catalysts, and creating more efficient exhaust gas treatment methods.

### Template-assisted method

Another approach to producing 1D materials is the template-assisted method. Here, the desired materials are infused into the pores of a template matrix, which is subsequently removed, leaving behind the 1D structures. These pores typically range in size from tens to hundreds of nanometers. Various porous materials can serve as templates, including porous zeolites [59], biomass materials [60], and porous alumina, commonly known as anodized aluminum oxide (AAO) [26]. Among these, AAO membranes are the most employed templates. The length of the nanostructures can be precisely tailored by adjusting the deposition time, ranging from a few to tens of micrometers, although this is constrained by the substrate thickness. This method offers a versatile means of fabricating 1D materials with controllable dimensions and properties.

**Figure 5.**
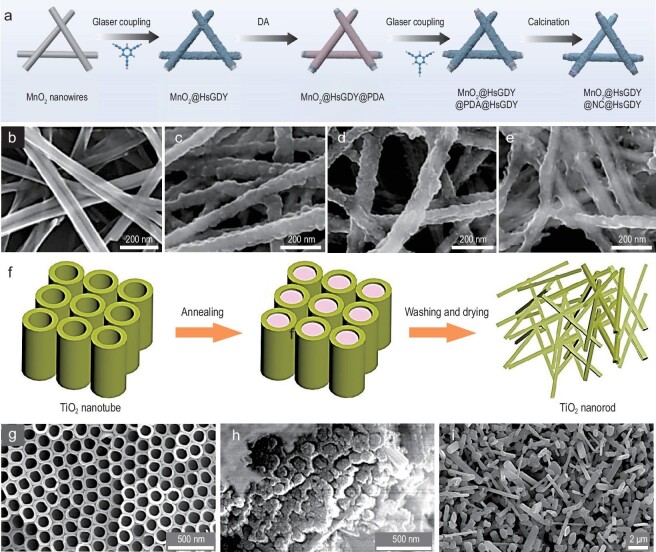
(a) Schematic diagram shows that the one-dimensional material of the MnO_2_@HsGDY@NC@HsGDY is obtained by using MnO_2_ nanowires as a template and sequentially coating hydrogen-substituted graphdiyne (HsGDY), polydopamine (PDA), and HsGDY shell layers. (b) SEM image of MnO_2_ nanowires. (c) SEM image of MnO_2_@HsGDY@PDA composites. (d) SEM image of MnO_2_@HsGDY@PDA@HsGDY samples. (e) SEM image of MnO_2_@HsGDY@NC @HsGDY samples. Adapted with permission from ref. [63]. Copyright 2024, Elsevier. (f) Schematic illustration of the fabrication of TiO_2_ nanorods. (g) SEM image of TiO_2_ nanotubes. (h) SEM image of NaCl@TiO_2_ nanotubes after annealing at 760°C. (i) SEM image of TiO_2_ nanorods. Adapted with permission from ref. [59]. Copyright 2021, Elsevier.

Currently, a series of dielectric, magnetic, and dielectric/magnetic composites have been synthesized using template-assisted methods, all of which exhibit high-performance EMW absorption properties [61,62]. For instance, MnO_2_@HsGDY@PDA@HsGDY nanowires were fabricated through successive coating of hydrogen-substituted graphdiyne (HsGDY), polydopamine (PDA), and HsGDY shells (Fig. 5a) using MnO_2_ nanowires as templates [63]. These 1D nanowires combine the advantages of multi-component and multi-core-shell (Fig. 5b–e). The enhanced electrical conductivity improves the dielectric loss of the material, while multiple heterogeneous interfaces within the structure lead to interface polarization. Both contribute to excellent EMW absorption performance with an RL_min_ of −68.5 dB and an EAB of 6.7 GHz. Furthermore, the template self-assembly technique has been utilized to grow rutile-type TiO_2_ nanorods on TiO_2_ nanotubes [59] (Fig. 5f–i). This method addresses the issue of poor response of TiO_2_ in the EMW region by utilizing the resonance polarization of ion clusters within rutile-type TiO_2_ nanorods, which enhances the charge storage capacity. EMW absorption tests indicate that frequency-selective absorption is achieved due to impedance matching anomalous resonance.

However, the template method presents some notable challenges. For instance, during the template removal process, structural damage or deformation may occur, thereby affecting the morphology and performance of the final product. As a well-thought-out example, Panina *et al*. utilized the AAO (anodized aluminum oxide) templates to fabricate various 1D nanostructures [26]. By adjusting the preparation parameters of the template, electrodeposition time, deposition voltage, and electrolyte solution, they could alter the composition, morphology, size, and electromagnetic parameters of the nanostructures. Subsequently, etching the AAO in a concentrated alkali solution yielded a series of 1D nanostructures with different pore sizes and densities, the parameters of which are tailored according to different etching processes. By the template-assisted method, dielectric composite TaC/C nanowires were prepared using a bamboo powder template, which volatilized organic gases at high temperatures for template removal [64]. The introduction of amorphous carbon into TaC nanowires effectively optimizes the anisotropy, thereby enhancing impedance matching. The amorphous carbon also introduces numerous defects and interfaces, further promoting dipole and interfacial polarization. The synergistic effect of multipolar loss and magnetic loss mechanisms significantly contributes the EMW attenuation performance. The TaC/C nanowires exhibit an RL_min_ of –54.2 dB and an EAB of 3.6 GHz at a thickness of only 1.4 mm.

The application of template-assisted methods in synthesizing 1D nanostructures is of great significance. However, the selection of template materials and preparation conditions poses challenges, which could constrain their applicability in 1D material synthesis due to their diverse characteristics and limitations. Additionally, template methods often entail multiple steps and intricate operations, leading to increased process complexity and cost. Therefore, a key current research focus is to explore cost-effective and non-toxic template removal methods to maintain product stability while trimming manufacturing expenses.

### Other methods

In addition to the methods mentioned above, the sol-gel method [65], low-temperature liquid phase reduction method [66], microwave heating synthesis technology [17], electroless coating technique [67], Friedel-Crafts alkylation [68], electroplating [69], arc discharge plasma process [70], and 3D printing technology [71] have been employed for manufacturing 1D nanomaterials.

Notably, the sol-gel method, as a convenient synthesis approach, has shown significant advantages in the preparation of 1D structured nanomaterials. This method allows for reactions at low temperatures, reducing potential material degradation or thermal decomposition issues associated with high-temperature environments, thus yielding higher purity products. Additionally, the sol-gel method provides a simple and controllable process for producing uniform nanostructures [72]. The low-temperature liquid phase reduction method is also a common approach for synthesizing 1D materials to avoid potential material degradation or thermal decomposition issues. The resulting products often possess higher purity. Compared to other synthesis methods, the low-temperature liquid phase reduction method offers relatively simple operation without the need for complex equipment and conditions. Additionally, magnetic field-assisted synthesis and 3D printing technology are innovative methods for the preparation of 1D nanomaterials. The magnetic field-assisted synthesis method adjusts the arrangement and self-assembly process of magnetic NPs by applying an external magnetic field, thereby regulating the morphology of the final 1D nanowires [73]. This method allows for precise control of the structure and properties of nanomaterials. On the other hand, 3D printing technology is known for its flexible, rapid, and highly customizable manufacturing process, which enables one-time shaping of complex structures, showing great potential [71]. These methods provide new insights and perspectives for the preparation of 1D EMW absorbing materials. With ongoing advancements in these technologies, they are expected to further drive the application of 1D nanomaterials in fields such as electronics, energy storage, and sensors.

## STRUCTURAL DESIGN OF 1D EMW ABSORBERS

The structural design of 1D EMW absorbers plays a crucial role in enhancing their absorption performance. Nanomaterials such as NFs, NWs, NCs, NTs, and NRs can be well engineered with many advantages that make them outstanding materials for EMW absorption. Advantages are not limited to the following: strong anisotropy, high conductivity, unique electromagnetic properties, long electron transmission paths, great surface area, strong interfacial polarization effect, excellent electromagnetic properties, etc. By well designing the morphology and structure, such as introducing hierarchical structures, constructing heterogeneous interfaces, and optimizing geometric parameters, superior impedance matching and enhanced EMW attenuation can be achieved.

Specifically, the introduction of hierarchical structures can create multi-level pores and channels within the 1D nanomaterials. This not only increases their specific surface area, but also provides more reflection and scattering paths for EMW absorption efficiency. In addition, the design of the heterogeneous interfaces helps the generation of multiple electromagnetic loss mechanisms within the 1D materials, including dielectric and magnetic losses, which further enhances EMW attenuation [34,74]. Optimizing geometric parameters, such as the length, diameter, and arrangement of the 1D nanomaterials, allows for optimal impedance matching, minimizing EMW reflection and maximizing absorption. Through these meticulous structural designs, 1D EMW absorbers can show exceptional performance in various application scenarios, offering broad development prospects and application potential.

### Nanofiber materials

**Figure 6.**
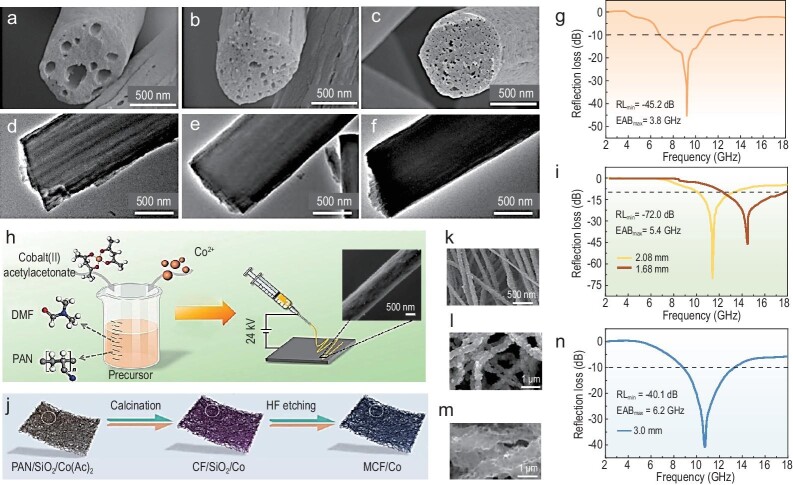
(a–c) SEM images and (d–f) TEM images of multichannel CNFs with different mass ratios of sacrificial agent to PAN (1:1, 2:1, and 3:1). (g) 2D RL curve of MC–CNFs-1:1–900 sample. Adapted with permission from ref. [75]. Copyright 2024, American Chemical Society. (h) Schematic illustration of the synthesis process of Co/C magnetic nanofibers using DMF (N,N-dimethylformamide) and PAN (polyacrylonitrile). Inset is the SEM image of a Co/C nanofiber sample. (i) 2D RL curves of the Co/C composites. Adapted with permission from ref. [80]. Copyright 2023, Elsevier. (j) Schematic illustration of the synthesis process of the MCF/Co composites. (k) SEM image of the PAN/SiO_2_/Co(Ac)_2_ composites. (l) SEM image of the CF/SiO_2_/Co composites. (m) SEM image of the MCF/Co composites. (n) 2D RL curve of the MCF/Co composites. Adapted with permission from ref. [82]. Copyright 2023, Elsevier.

Nanofiber materials, including carbon nanofibers (CNFs), magnetic fibers, and magnetoelectric composite fibers, have recently garnered significant attention in the field of EMW absorption. Among these, CNFs have emerged as ideal lightweight and efficient EMW absorbing materials due to their high aspect ratio, low density, adjustable diameter, and excellent dielectric loss and electrical properties. The dielectric loss of CNFs primarily depends on their conductivity and polarization loss. Combined effects of dipole orientation loss and interfacial polarization loss, caused by uneven distribution between different media, enable CNFs to exhibit superior EMW absorption performance [75,76]. More recently, Li *et al*. [75] prepared lotus-root like multichannel CNFs through sacrificial template-assisted electrospinning, followed by traditional stabilization and carbonization (Fig. 6a–f). The channel structure can be regulated by adjusting the sacrificial agents. Multichannel construction provides multi-reflection and scattering to prolong the propagation path of EMW, thereby improving dissipation efficiency (Fig. 6g).

Other 1D nanofibers, such as SiC [9], SiCN [75], and NbN [46], have also attracted considerable attention. These 1D nanomaterials possess unique chemical compositions and crystal structures, which provides more options and possibilities for the design and fabrication of EMW absorption materials. For instance, peanut shell-like SiC nanofibers exhibit an RL_min_ of up to 48 dB at 11.1 GHz, with an EAB covering most of the X-band [9]. Meanwhile, SiCN nanofibers synthesized by Xiao *et al*. offer adjustable dielectric constants and excellent thermal stability, showing promising application prospects in the field of EMW absorption. The fibers exhibited an RL_min_ value of −31.1 dB at a thickness of 2 mm and an EAB of 4.59 GHz [77]. Additionally, niobium nitride nanofibers present new possibilities and avenues for applications in EMW absorption due to their excellent conductivity [46]. The high aspect ratio of magnetic nanofibers implies strong anisotropy, which can enhance EMW absorption performance. Among all magnetic fiber materials, Fe nanofibers [78] and Cu nanofibers [79] stand out for their excellent dielectric constants and magnetic permeabilities. They become promising candidates with facilitated eddy currents and dielectric losses.

Undoped dielectric nanofiber materials exhibit relatively weak capabilities in EMW absorption. In contrast, composite magnetic materials, especially comprising bimetallic alloy magnetic particles [80,81], oxides [82], and ferrites [80] with good soft magnetic properties, represent effective approaches for improving performance. Composite magnetic materials have many excellent properties, such as abundant heterointerfaces, optimized defect levels, and established magnetic coupling networks. The prime example is the study reported by Ma and co-workers, where Co NPs were introduced to improve the magnetic loss ability of CNFs by the former route [80]. As shown in Fig. 6h, Co NPs are reduced *in-situ* and encapsulated in the fibers. Leveraging the synergistic effects of microstructure, component, and dimension, the microporous composites provide an RL_min_ of −72.0 dB (Fig. 6i).

Nitrogen-doped carbon nanofibers decorated with nanoscale SnFe_2_O_4_ particles can be synthesized using a combination of electrospinning and solvothermal treatment [83]. The prepared lightweight materials exhibit strong absorption at a thickness as thin as 1.6 mm. The introduction of magnetic compositions not only contributes to enhance the permeability of materials, but also optimize the defect level within the composites. Moreover, the combination of 1D hollow carbon fibers with CoFe_2_O_4_ hollow NPs produces composites with distinct sizes and loss mechanisms [7]. By adjusting the decomposition temperature, the EM parameters can be fine-tuned. Yang and co-workers reported an example of a design strategy that combined dual dielectric and magnetic loss mechanisms. One-dimensional hollow carbon fibers were assembled with CoFe_2_O_4_ hollow NPs to create composites with distinct sizes and loss mechanisms [7]. The RL_min_ of the 1D composite reaches −57.8 dB at 14.8 GHz, with an EAB of 5.9 GHz, covering nearly the entire Ku-band.

Wu's group dispersed the double-shell Ni@SiC@C NPs into 1D carbon nanofibers to solve the impedance mismatch [84]. Owing to the 3D conductive network and enriched heterointerfaces, the obtained composites achieved a strong absorption of −-53.3 dB, coupled with a remarkable absorption bandwidth of 15.47 GHz. Yang *et al*. [82] employed hard-template–assisted electrospinning to fabricate 1D CFs (MCF/Co) modified with magnetic metal NPs (Fig. 6j–m). The hollow structure of composite nanofibers leads to strong interfacial polarization losses and multiple reflection effects. The level of impedance matching is further enhanced by the introduction of cobalt NPs due to magnetic losses. Consequently, MCF/Co nanofibers exhibit broad EAB (6.2 GHz) and robust EMW absorption (−40.1 dB, Fig. 6n). These studies demonstrate that EMW wideband absorption can be achieved by adjusting the structure and composition, such as introducing micro- and nano-scale magnetic particles into dielectric materials.

Recently, high entropy (HE) materials with a high degree of compositional complexity have become emerging functional materials. The efficient synergism among multiple components, tunable electronic structures, and unique dielectric/magnetic characteristics of HE materials have attracted extensive attention in the realm of EM protection [85–87]. However, their further development is hampered by high density and poor chemical stability. The integration of HE components into 1D carbon nanofibers is one of the promising approaches to solve the above issues. As a case in point, Shen *et al.* [88] employed the microwave-induced carbon thermal shock (MCTS) technique to prepare FeCoNiAlMn high-entropy alloy (HEA) NPs-modified CFs. It exhibited the strongest absorption with an RL_min_ value of −63.6 dB at 2.4 mm thickness and EAB of 6.67 GHz at a thickness of only 1.9 mm. In a very recent study, Wang *et al.* [89] fabricated FeCoNiCuZn HEA NPs anchored on CFs. The NPs delivered satisfactory low-frequency MA performances. Such excellent EM attenuation ability is attributed to several factors, including the high conductivity of the 3D network established by 1D CFs, high configurational entropy effects, and optimized defect levels along the HEA-carbon heterointerfaces. Based on the above insights, combining HE materials with 1D CFs has led to favorable physicochemical, electrical, and magnetic properties that can enhance EMW absorption performances.

Surface modification of nanofibers prior to use is one of the most promising approaches for enhancing performance. Dielectric loss in CNFs primarily arises from conductivity and polarization losses, while the absorption performance of magnetic NFs is closely related to their magnetic permeability. Additionally, morphological control and added special magnetic materials can induce both magnetic and dielectric losses, thereby optimizing the nanofibers’ structure, diameter, and magnetic composition. These measures collectively contribute to improving the overall EMW absorption performance of nanofibers.

### Nanowire materials

Combining unique morphologies with multiple components has proven to be an effective strategy for enhancing EMW attenuation. The 1D nanowire materials provide excellent electron transport paths, and the close spacing between nanowires facilitates the formation of conductive networks, leading to significant conductive losses. Additionally, the heterogeneous structure offers multiple reflection interfaces, allowing EMWs to undergo repeated reflections and scattering within the nanowire. For instance, 1D MoO_2_/N-doped carbon nanowires with heterogeneous structures were synthesized via a mild *in-situ* chemical oxidative polymerization followed by carbonization [90]. Experimental results show that this unique structure enables the composites with a filler content of 40 wt% and a thickness of only 2.3 mm, to achieve an RL_min_ of −35.0 dB at 8.37 GHz. Currently, silicon carbide (SiC) is widely utilized as a broadband semiconductor material in the realm of high-temperature and wide-frequency EMW absorption. SiC nanowires, accompanied by abundant stacking faults during growth, can aggregate a large number of holes and electrons, thereby enhancing dielectric loss. The presence of vacancies and dipoles can further increase the polarization losses. In another work, Dou and co-workers controlled over the structure of SiC nanowires through carbon-thermal reduction, thereby adjusting electromagnetic parameters [91]. The generation of defects and the modulation of morphology facilitate the strong absorption of EMWs. Other reported 1D dielectric nanowire materials include MoO_2_/NC [90], MnO_2_ [92], etc.

**Figure 7.**
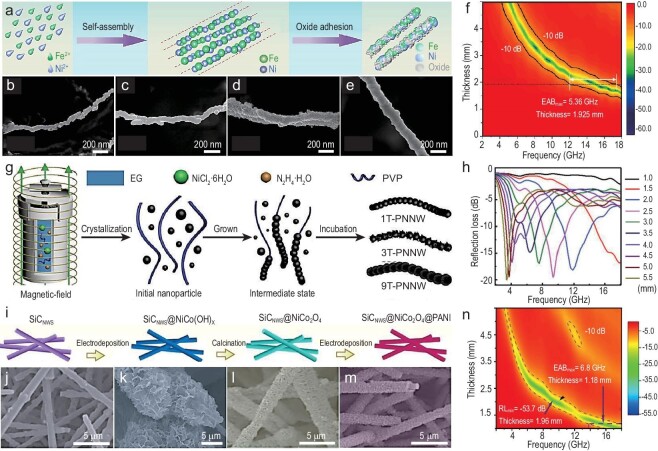
(a) Schematic diagram of synthesis of Fe–Ni composites. (b–e) SEM images of Fe-Ni composites with different molar ratios of Fe and Ni (2:1, 1:1, 2:3, and 1:2). (f) Mapping of RL versus the test frequency and thickness of the EMW absorber, Fe–Ni (1:2) nanowires. Adapted with permission from ref. [93]. Copyright 2023, American Chemical Society. (g) Schematic diagram of the synthesis of PVP-directed nickel nanowires (PNNWs) under different magnetic field strengths (1T, 3T, and 9T). (h) RL curves of 3T-PNNWs. Adapted with permission from ref. [94]. Copyright 2022, Wiley. (i) Schematic illustration of the preparation process of SiC_NWS_@NiCo_2_O_4_@PANI (polymer polyaniline) 1D hierarchical nanocomposites. (j) SEM image of SiC_NWS_ sample. (k) SEM image of SiC_NWS_@NiCo(OH)_x_ sample. (l) SEM image of SiC_NWS_@NiCo_2_O_4_ sample. (m) SEM image of SiC_NWS_@NiCo_2_O_4_@PANI nanocomposite. (n) RL curve of SiCNWS@NiCo_2_O_4_@PANI nanocomposite. Adapted with permission from ref. [74]. Copyright 2022, Elsevier.

Magnetic nanowire absorbers, due to their strong anisotropy, generate a vector superposition effect of electric/magnetic field distribution for EMW absorption. In a recent study, Yang *et al*. [93] prepared Fe-Ni heterogeneous nanowires using magnetic field-assisted liquid-phase reduction (Fig. 7a). By adjusting the composition ratio of Fe and Ni, the electromagnetic parameters of 1D nanowires can be effectively controlled (Fig. 7b–e). When the ratio of Fe to Ni is 1:1.5, the nanowires exhibit optimal EMW absorption properties, with an RL_min_ of up to −26.1 dB, while the EAB covers the Ku band (Fig. 7f). In another example, Ni nanowires with high aspect ratio, large surface area, and excellent magnetic properties were synthesized using a magnetic-induced solvothermal method [94], as illustrated in Fig. 7g. Various electromagnetic loss mechanisms are induced to attenuate EMWs, resulting in an RL_min_ of −29.8 dB at 4.0 GHz, with an EAB of 3.6 GHz (Fig. 7h). The surface of the nanowires contains a large number of disordered atoms and vacancies, which easily induce a localized crystal field perpendicular to the surface. This specific direction of the crystal field tends to create anisotropy. Additionally, the large surface area of magnetic NPs implies strong interfacial polarization effects. Therefore, magnetic nanowires are a highly promising material for EMW absorption. Although 1D magnetic nanometals possess high saturation magnetization and Curie temperature, their ability to attenuate EMW is limited by the Snoek law [95]. This constraint causes a rapid decline in magnetic permeability at high frequencies, thereby reducing their EMW absorption performance.

Composite dielectric and magnetic materials can achieve a synergistic effect in EMW performance, resulting in a ‘1 + 1 > 2’ effect. As illustrated in an early work, Wu *et al*. synthesized FeB/B_4_C nanowire composites using a catalytic-assisted process, achieving electromagnetic absorption across multiple bands [96]. The abundant defects and interfaces in B_4_C induce strong relaxation losses, while the magnetic FeB NPs contribute to magnetic losses at high frequencies. In another example, the SiC NWS@NiCo_2_O_4_@PANI hierarchical composite, composed of dielectric SiC, magnetic NiCo_2_O_4_, and conductive polymer PANI (polymer polyaniline), was fabricated on the surface of SiC nanowires via a two-step method [74] (Fig. 7i–m). The excellent impedance matching enables the nanowire to achieve an RL_min_ value of −53.7 dB and an EAB of 4.16 GHz (Fig. 7n). In recent years, the improvement of EMW absorption performance of nanowire materials through morphology control has garnered significant research attention. According to the recent research by Yang *et al*. [13], the introduction of a core-shell structure not only enhances interfacial polarization loss, but also improves the impedance matching performance of nanowires. They showed that Fe@PANI core-shell nanowires prepared via a one-pot method have an absorption performance of −31.8 dB. In another recent report, core-shell heterogeneous Fe NWs@SiO_2_ nanostructured composites were synthesized via a simple liquid-phase hydrolysis reaction [97]. Attributed to the EM loss of composites, the small-scale effects induced by the 1D nanostructure, as well as the polarizing effect at the core-shell heterogeneous interface, the RL_min_ reaches −54.8 dB. By further optimizing the structure and composition of nanowires, their performance can be improved, thereby expanding their application range across different frequency bands.

Additionally, combining nanowires with other functional materials such as magnetic and dielectric materials can enable multifunctional composite absorbers to meet diverse demands in various fields. The combination of unique morphologies and multi-component designs can significantly enhance the EMW absorption performance of nanowire materials. Strategies such as surface modification, morphological control, and the incorporation of magnetic or dielectric materials have been shown to improve absorption capabilities of nanowires and broaden their application potential. Future research should focus on optimizing nanowire structures and compositions to achieve even better performance and wider applications.

### Nanochain materials

**Figure 8.**
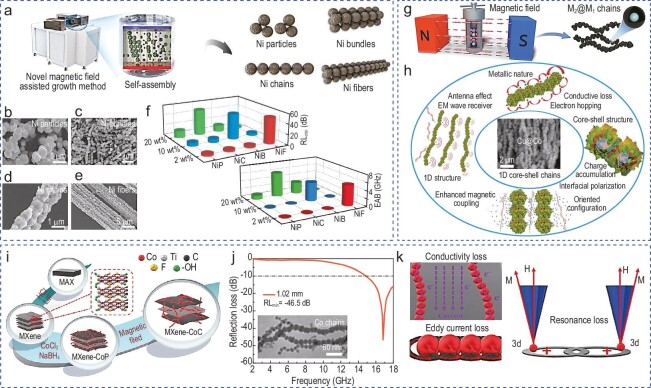
(a) Illustration of the preparation process of Ni-based MAs (microwave absorbers) with different sequential architectures assisted by magnetic field. (b) SEM image of Ni particles. (c) SEM image of Ni bundles. (d) SEM image of Ni chains. (e) SEM image of Ni fibers. (f) RL_min_ and EAB values of four samples with different filling ratios from matching thicknesses of 0.5 to 5.0 mm. Adapted with permission from ref. [98]. Copyright 2023, Wiley. (g) Schematic illustration showing the fabrication process of the core-shell magnetic chains. (h) EMW absorbing mechanism of the core-shell magnetic chains. Adapted with permission from ref. [99]. Copyright 2020, Wiley. (i) Illustration of the fabrication process of the MXene-CoC (Co chain) composites. (j) Reflection loss of MXene-CoC composites. Inset is the SEM image of Co chains. (k) EMW absorbing mechanism of MXene-CoC composites. Adapted with permission from ref. [101]. Copyright 2021, Elsevier.

Nanochain materials are typically formed through the self-assembly and interconnection of metal or metal oxide particles, with lengths ranging from nanometers to micrometers. By controlling the orientation and aspect ratio of the NPs, the complex permittivity and complex permeability of the material can be adjusted. As a result, the impedance matching of 1D materials is optimized and the effective absorption bandwidth is expanded. As early as 2016, nanochains composed of paramagnetic FeCo NPs were synthesized by Zhang and co-workers using the surface wet chemical method [22]. The asymmetric structure resulting from the 1D chain assembly of cubic FeCo particles surpasses the Snoek limit and induces strong anisotropy. The magnetoelectric synergy enables the FeCo nanochains to exhibit an absorption bandwidth of < −20 dB in the 2∼10 GHz range. However, traditional nanochain absorbers with high fill ratios struggle to meet the lightweight requirements of modern applications. Recent advances in 1D nanochains have addressed this issue by designing specific spatial layouts to regulate the microstructure. When functional units (NPs) form an ordered 1D structure, the magnetic coupling, magnetic anisotropy, and magnetic permeability between the units are enhanced. Concurrently, significant domain motion phenomena emerge and become more frequent as the length of the ordered structure increases. This enhanced domain motion helps to respond to incident microwaves, thereby increasing energy storage and loss capacity. As a consequence, the material's macroscopic properties are improved. As shown in Fig. 8a, Pan and co-workers employed a magnetic field-assisted growth method to fabricate nickel chains (NiCs) with a bottom-up ordered structure [98]. The electromagnetic parameters and domain motion of nanowires can be precisely controlled through spatial engineering (Fig. 8b–e). The synthesized chains exhibit unique electromagnetic behaviors, such as the generation of negative permittivity and anisotropic chiral vortices, which enhance the absorption performance of nanochains at low fill ratios (Fig. 8f). To change the orientation of the 1D magnetic chains, Che *et al*. [99] constructed a 1D core-shell bimetallic magnetic nanochain (Fig. 8g) and improved the EMW absorption performance by adjusting the complex permittivity and permeability. Additionally, the formation of a multi-tiered core-shell structure facilitates the creation of multiple interfaces, thereby augmenting magnetic coupling interactions and polarization loss (Fig. 8h). The directed Cu@Co nanowires achieve an optimal RL_min_ of −43.5 dB and an EAB of 7.3 GHz. Another example is an absorber composed of Fe@ZrO_2_ nanochains, which is fabricated via the arc discharge plasma method [70]. The high dielectric constant ZrO_2_ shell reduces the interference of the intrinsic magnetism of the Fe core, facilitating the establishment of strong magnetic/dielectric coupling within the nanochains. Owing to the high multiple resonances and proper matching, the nanochains achieve an RL_min_ value of −45.3 dB at a thickness of 3 mm.

Generally, single magnetic materials face challenges in achieving strong absorption in broadband due to their low Curie temperature (resulting in loss of magnetism at high temperatures) and impedance mismatch. Therefore, composite dielectric materials have become a hot topic in the research of absorbing materials [61]. A novel synthesis method has been reported to prepare tunable 1D Co@PANI nanochains by reducing metal ions under a parallel magnetic field, pretreating with KH550, and pre-oxidizing [100]. By altering the metal elements, the impedance matching was adjusted, resulting in an RL_min_ of −73.1 dB at 4.6 mm for the nanochains. This significant absorption intensity is ascribed to the synergistic effects of dielectric and magnetic losses.

Designing multi-interface or layered structures is an inevitable trend in the current development of electromagnetic materials. Pan and co-workers synthesized a heterostructure composed of Ti_3_C_2_T_x_ MXene and 1D Co nanochains through a simple *in-situ* process, as illustrated in Fig. 8i [101]. At 16.7 GHz, the MXene/Co nanochain composites achieved an RL_min_ of −46.5 dB, with a matching thickness of only 1.0 mm (Fig. 8j). The electromagnetic attenuation mechanism of nanochain composites primarily includes interface polarization loss and magnetic loss. Interface polarization loss is caused by the accumulation of space charge at the heterogeneous interface. Magnetic loss, on the other hand, results from domain motion and changes in magnetic flux due to the anisotropic characteristics of the magnetic chains and the cross-linking magnetic field effect (Fig. 8k). The results indicate that absorbers with magnetoelectric synergy can achieve electromagnetic balance, optimize impedance matching, and enable precise control over multiple functionalities. In this way, new avenues and possibilities are provided for the design and application of absorptive materials.

By precisely controlling the orientation and aspect ratio of NPs, as well as designing core-shell structures with specific configurations, researchers can optimize the material's dielectric constant and magnetic permeability. These improve impedance matching and extend the effective absorption bandwidth. As a result, the EMW absorption performance of nanochain materials can be significantly enhanced over a broad frequency range. Future research should continue to explore the structural design and functional tuning of nanochain materials to achieve even broader applications and superior performance.

### Nanotube materials

**Figure 9.**
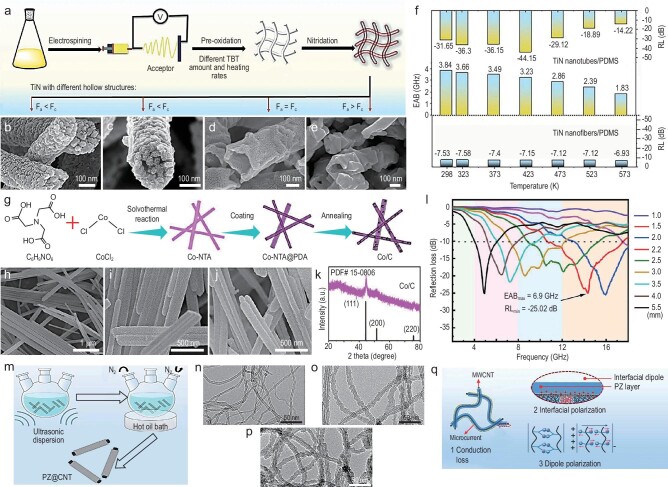
(a) Schematic illustration of the fabrication process of TiN nanotubes. (b–e) SEM images of TiN nanotubes with different hollow structures containing different tetrabutyl titanate (TBT) amounts and heating rates of pre-oxidation under air atmosphere: (b) 5.05 mL, 2°C min^−1^, (c) 3.8 mL, 2°C min^−1^, (d) 3.8 mL, 1°C min^−1^, and (e) 3.8 mL, 0.5°C min^−1^. (f) EAB and RL_min_ values of TiN nanotubes/PDMS (polydimethylsiloxane) and TiN nanofibers/PDMS. Adapted with permission from ref. [102]. Copyright 2024, Springer. (g) Synthetic process diagram of Co/C nanotubes. (h) SEM image of cobalt-nitrilotriacetic acid (Co-NTA) samples. (i) SEM image of Co-NTA@PDA (polydopamine) samples. (j) SEM image of Co/C nanotubes. (k) XRD pattern of Co/C nanotubes. (l) RL values of Co/C nanotubes. Adapted with permission from ref. [103]. Copyright 2024, American Chemical Society. (m) Schematic fabrication process of polyzwitterion wrapped CNT (PZ@CNT). (n–p) SEM images of PZ@CNT-x (x = 1, 2, and 3, sample name) with different molar ratios of ViIMSO_3_ (1-vinyl-3-imidazole-propane sulfonate) and DVB (divinylbenzene) for the preparation of samples: (n) 9 mmol, 4.5 mmol; (o) 18 mmol, 9 mmol; and (p) 36 mmol, 18 mmol. (q) EMW absorbing mechanism of encapsulated carbon nanotube (PZ@CNT). Adapted with permission from ref. [104]. Copyright 2022, Elsevier.

Achieving broadband absorption in EMW absorbing materials is challenging due to the inherent properties, frequency dependency, structure, design aspects, and other factors. Nanotube materials, with high aspect ratio, excellent conductivity, large specific surface area, and ease of functionalization, excel in absorbing EMWs across various frequency bands. Furthermore, the electronic properties of nanotubes, determined by their morphological structure, allow for diverse electromagnetic response characteristics, enabling broadband absorption. Gong *et al*. [102] showed an example where well-engineered nanotube structures can be optimized to manipulate dielectric losses and impedance matching (Fig. 9a). TiN nanotubes, known for their electromagnetic coupling effects, good conductivity, high melting point, environmental stability, and rich interface characteristics, were synthesized by controlling the diffusion kinetics and the Ostwald ripening process. When combined with polydimethylsiloxane (PDMS), these nanotubes form enhanced heterointerfaces (Fig. 9b–e) that generate increased polarization relaxation losses, thereby improving EM absorption efficiency and optimizing impedance matching at high temperatures. Consequently, the composites exhibit an RL_min_ of −44.1 dB across a temperature range of 298∼573 K, albeit with a narrow absorption bandwidth of 3.2 GHz (Fig. 9f).

By designing and optimizing CNT structures and composites, effective broadband EMW absorption can be achieved. For example, N-doped Co/C nanotubes with various morphologies have been synthesized using solvothermal and pyrolysis processes [103] (Fig. 9g–k). Adjusting the ratio of cobalt salt to solvent results in a absorption of −25.0 dB at 2.2 mm and an EAB of 6.9 GHz (Fig. 9l). The enhanced EMW absorption performance is attributed to the 1D nanotube structure, which facilitates the formation of 3D conductive networks. Impedance matching is optimized, and the attenuation capabilities are enhanced.

Nanotube materials can be integrated with various base materials to optimize their structure and composition to achieve a suitable impedance matching. This enables strong absorption of EMWs across a broader frequency spectrum. Yang *et al*. [104] utilized poly (1-vinyl-3-methylimidazolium propane sulfonate) (PZ) to encapsulate carbon nanotubes (Fig. 9m). A straightforward *in-situ* polymerization method was used to synthesize the core-shell structure of dual-ionic encapsulated carbon nanotubes (PZ@CNT), addressing the issue of impedance mismatch caused by the high conductivity of nanotubes (Fig. 9n–p). This innovative approach yielded remarkable results, achieving an RL_min_ of −44.5 dB and an EAB of 4.2 GHz, surpassing the performance level of previously reported single nanotube materials [105] (Fig. 9q). Additionally, nitrogen-doped carbon nanotubes combined with CoNi nanocomposites form a layered microstructure of porous CoNi@N-doped carbon nanotubes. This composite achieves an RL_min_ of up to −64.5 dB and EAB of 3.9 GHz at loading as low as 10 wt% [106]. To achieve a moderate dielectric constant, Xiong *et al*. combined imidazolium-based ionic liquids (IMILs) with CNTs to prepare composites through a simple mixing process [107]. Compared to pure CNT, the IMIL/CNT composites show better impedance matching, which facilitates the efficient entry and attenuation of EMWs within the composites. This composite not only achieves an RL_min_ of −46 dB, but also exhibits EAB up to 4.8 GHz.

Currently, MOFs are widely utilized in the field of EMW absorption due to their porous structure, ultra-large surface area, easy functionalization of building units, controllable assembly, diverse morphologies, as well as high porosity. The combination of MOFs with nanotubes not only enhances their dispersibility but also further strengthens their microwave absorption capability by forming new interfaces and electron transport channels. This is demonstrated by a CNT/FeCoNi@C composite composed of magnetically derived MOF nanospheres and Fe-filled CNT sponges. The composite exhibits ultra-low density, relatively high specific surface area, and outstanding stability [108]. It was observed at −51.7 dB for RL_min_ and 6.0 GHz for EAB. MOFs in the composite are primarily converted into nanostructured functional materials through thermal treatment. It leads to various metal ion valence states and interface polarization, thereby achieving EMW absorption.

Although nanotube EMW absorbing materials demonstrate excellent performance, improvements are still needed in areas such as absorption frequency range, absorption efficiency, material stability, manufacturing process, mechanical properties, environmental friendliness, and composite design. Some strategies, such as optimizing the material structure, simplifying the manufacturing process, enhancing environmental adaptability and mechanical strength, and developing green composites are expected to further enhance their application potential and reliability in military, communications, electronic devices, and other fields.

### Nanorod materials

Compared to nanofiber materials, nanorods typically have a smaller aspect ratio, making it more challenging to construct a 3D interconnected conductive network. Consequently, 1D nanorod materials are usually regarded as short dipole antennas, with dielectric losses primarily contributed by polarization relaxation at their internal dipoles and heterogeneous interfaces.

**Figure 10.**
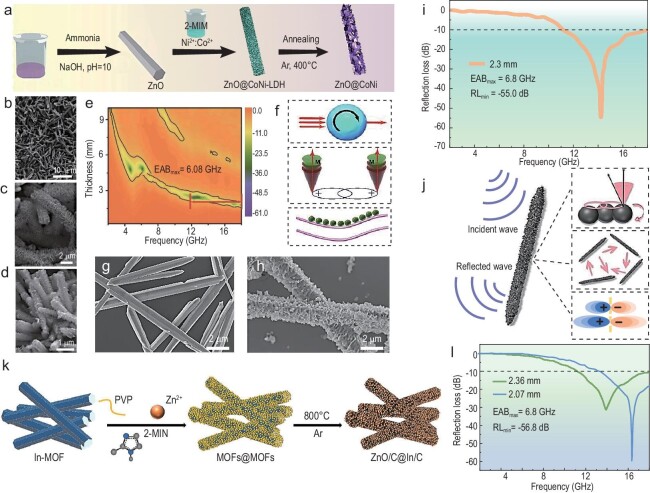
(a) Illustration of the synthetic process of NiCo@C/ZnO nanorods. (b) SEM image of ZnO nanorods. (c) SEM image of ZnO@NiCo-LDH (layered double hydroxides) composites. (d) SEM image of NiCo@C/ZnO composites under air atmosphere. (e) Reflection loss value of NiCo@C/ZnO nanorod-like composites. (f) EMW loss mechanism of NiCo@C/ZnO nanorods. Adapted with permission from ref. [109]. Copyright 2021, Springer. (g) SEM image of ZnO nanorods. (h) SEM image of ZnO@CoNi/C composites. (i) Frequency dependence of RL curve of ZnO@CoNi/C composites. (j) Microwave absorption mechanism of ZnO@CoNi/C 1D materials. Adapted with permission from ref. [110]. Copyright 2024, Elsevier. (k) Schematic of the preparation process of ZnO/C@In/C hetero-nanorods with a core-shell structure. (l) RL curves of ZnO/C@In/C core-shell heterorods. Adapted with permission from ref. [114]. Copyright 2023, Elsevier.

Nanorods are typically prepared using *in-situ* assembly and MOF-derived synthesis methods. These synthesis processes involve dielectric and magnetic components, where the combination of two types of components can effectively balance the relationship between the dielectric constant and magnetic permeability. Then better impedance matching is possible. Optimized impedance matching induces magnetoelectric coupling effects, further enhancing the attenuation capacity of EMWs. This is illustrated by the design of NiCo layered double hydroxides@ZnO nanorod materials with a heterogeneous structure using hydrothermal methods and then obtaining NiCo@C/ZnO composites by pyrolysis (Fig. 10a–d) [109]. The composites exhibit outstanding absorption performance due to the synergistic effects of dielectric loss and magnetic loss. The magnetic coupling 3D network generated by NiCo NPs embedded in the 1D dielectric matrix can match the wavelength of the incident wave, interacting strongly with EMWs. At a matching thickness of 2.3 mm, the material shows absorption capability with an impressive RL_min_ of −60.9 dB. Besides, the composites exhibit a broader EAB, reaching 6.08 GHz (Fig. 10e, f). More recently, Wang *et al*. synthesized ZnO@CoNi/C nanorods using size design and heterogeneous interface engineering [110]. Briefly, ZnO nanorods were combined with CoNi nanosheets via a hydrothermal method. Subsequent encapsulation with polydopamine resulted in a unique 1D core-sheath structured ZnO@CoNi/C composite (Fig. 10g, h). After calcination, defects and multiple heterogeneous interfaces are exhibited, enhancing multiple scattering and reflection of EMWs upon penetration. The 1D architecture also facilitates electron hopping and migration, boosting the attenuation absorption. Remarkably, with a filling rate of only 20 wt%, the 1D nanorod composite observed an RL_min_ of −55.0 dB at a thickness of 2.3 mm, accompanied by an EAB value of 6.8 GHz (Fig. 10i, j).

In the recent decade, MOF-derivatization has become a crucial method for preparing 1D nanorod materials due to its numerous advantages [111]. Obviously, the synthesis method is simple, and the pore structure, pore volume, and particle size are easy to control. In addition, MOF materials can incorporate and polymerize other substances for multifunctional composites. Notably, various MOFs with different compositions and topologies can be converted into target materials, and the carbon matrix from MOFs’ pyrolysis prevents particle aggregation and structural collapse. These materials can retain original morphology after processing. Currently, there are emerging examples of MOFs being transformed into nanostructured functional materials for EMW absorption, such as porous carbon, metal oxides, metal sulfides, and their composites. Magnetic metal oxides can be generated *in-situ* on MOF-derived porous carbon surfaces or within their pores, achieving both dielectric and magnetic losses. Design strategies for MOF-derived nanomaterials include using MOFs as templates for composites, introducing heteroatoms to alter composition and morphology. There are also composites that use MOF with other materials as self-sacrificial templates. These strategies have been shown to enhance dielectric loss, magnetic loss, impedance matching, and attenuation performance.

The microstructure and conductivity of MOF-derived materials can be controlled by varying the carbonization temperature, thereby achieving tunable EMW absorption. One of the early examples is reported by Wu and co-workers, which used iron-based MOFs (Fe-MIL-88A) to synthesize porous rod-shaped composite nanomaterials [112]. The thermal decomposition temperature of the MOF precursor was found to be critical for forming ferromagnetic metal NPs and carbon matrices in the porous rod-shaped composites. The composites pyrolyzed at 600°C exhibited an RL_min_ of −52.9 dB with an absorber thickness of 2.5 mm, and an EAB of 4.6 GHz, covering the entire X-band. Soon after that, Hu *et al*. [14] reported the preparation of hollow FeNi/NiFe_2_O_4_ co-doped carbon composite nanorods (derived from FeNi-MIL-101) by a two-step process of solvothermal and pyrolysis treatments. The electromagnetic parameters and absorption performance of the resulting composites can be tuned by simply altering the calcination temperature. When calcined at 700°C, the FeNiC composite gives optimal EMW absorption performance, with an RL_min_ of −62.7 dB and a corresponding absorption bandwidth of 7.46 GHz, covering the entire Ku-band.

Constructing composites with multiple components has proven to be an effective approach for achieving impedance matching and strong attenuation capabilities. Consequently, the ‘MOFs + MOFs’ strategy has emerged. Using this strategy, 1D Mo_2_C/Co/C nanorods were synthesized [113]. Initially, 1D Mo-MOFs were fabricated via reflux heating. ZIF-67 was then employed to tailor the structure of 1D Mo-MOFs to generate the precursor Mo-MOFs/ZIF-67. Ultimately, Mo_2_C/Co/C ternary composite nanorods were synthesized through pyrolysis in an Ar atmosphere. This multi-component system not only optimizes impedance matching and forms rich heterogeneous interfaces, but also provides multiple channels for attenuating EMWs. Then, nanorods exhibit an RL_min_ of −54.6 dB at 16.2 GHz, with a corresponding thickness of 1.77 mm. In another work, Zhang *et al*. [114] proposed to devise a dual-MOF system with a core-shell structure by encapsulating ZIF-8 (Zn) within MIL-68 (In), from which core-shell porous carbon heterostructures were synthesized via pyrolysis (Fig. 10k). As the proportion of MIL-68 and ZIF-8 in the ZnO/C@In/C heterostructure nanorods is adjusted, impedance matching is achieved. Most EMWs are promoted into the composites. The nanorods attain an RL_min_ of −56.8 dB at 16.3 GHz and a broad EAB of 6.8 GHz at a thickness of 2.36 mm, attributed to the high attenuation capability arising from numerous interfaces (Fig. 10l). Using ‘MOFs + MOFs’ composites as sacrificial templates enriches the hierarchical structure of absorptive materials, allowing for the design of various microscopic structures and multiple interfaces. By optimizing EM parameters, this approach helps enhance electromagnetic response characteristics and promotes EMW dissipation. It is hoped that these material design concepts will guide the future development of absorbing materials.

## CONCLUSIONS AND PERSPECTIVES

Over the past decade, 1D nanostructures have been shown to have great potential advantages in the fabrication of EMW absorbing materials. Many studies have found that 1D structures possess novel physical properties and controllability, and significant progress has been made in a systematic investigation into the synthesis methods. The main advantages of unique electromagnetic properties, long electron transmission paths, high surface area, strong interfacial polarization effects, strong magnetic anisotropy, and easy controllability and functionalization have been summarized. The key to the enhanced attenuation mechanism of 1D nanostructures lies in the precise control of the electromagnetic parameters and magneto-dielectric coordination effects by adjusting their compositions, aspect ratios, and morphologies. Moreover, the optimization of these control parameters can enhance the attenuation of EMWs and meet specific application requirements by extending the charge transport path and promoting multiple scattering and reflections of EMWs.

Major advances and prominent advantages of different 1D nanomaterials, such as nanofibers, nanowires, nanochains, nanotubes, and nanorods, are summarized, laying the foundation for future research on 1D absorbing materials. Different 1D structures have their unique performance in attenuating the energy of EMWs. Hollow porous nanotubes reduce the density of the absorbing materials while increasing the multiple reflections and scatterings of EMWs. Similarly, the irregular growth of nanochain surfaces forms a 1D flower-like structure, significantly increasing the specific surface area. Furthermore, constructing heterogeneous structures such as core-shell structures, hollow structures, and multilayer structures can produce spatial charge polarization through interfacial effects. These designs significantly enhance the magnetic permeability and dielectric performance of 1D nanomaterials. Various loss mechanisms are introduced to optimize impedance matching, achieving efficient absorption over a wide frequency band. On the other hand, the limitations of single 1D dielectric or magnetic materials caused by a single electromagnetic loss mechanism and impedance mismatch restrict their performance. However, by functionally combining dielectric and magnetic materials through composites, the maximum penetration of EMWs into the material can be promoted, leading to new EMW attenuation enhancement processes. Indeed, the understanding of the interaction mechanism between EMWs and nanomaterial microstructures is critical for developing new design ideas in manufacturing novel high-performance and lightweight absorbers.

In general, traditional EMW absorbing materials are often made by mixing absorbers with epoxy resin or paraffin. While such approaches can leverage the advantages of the materials, it often overlooks the crucial role of structure in attenuating EMWs. To improve future absorptive materials, both material properties and structural design must be considered. A strategy that refines the absorbent materials into functional units can arrange them in an orderly structure. This approach ensures that both the materials and structural characteristics are fully exploited, thereby enhancing the absorption performance. To provide a deeper theoretical basis for materials development, exploration of the coupling mechanism of the materials’ sequence structures is needed. However, this research area is still in its early stages. There are many challenges that must be overcome in order to prepare efficient materials and construct the physical models. Building upon the current exploration, the following points are expected to aid future research:

- Starting from the particle nature of EMWs, the interaction mechanisms between EMWs and the microstructures needs to be further clarified, and the potential relationship between absorption frequency and electronic and charge movements should be determined.
- The impedance matching and synergistic effects between various components are worth investigating, and advanced characterization techniques and theoretical calculations can be utilized to reveal the mechanisms of EM loss.
- The intrinsic principles of 1D nanostructures on the propagation and attenuation of EMWs, as well as the influence of 1D morphology on EMW absorption, need to be elucidated.
- Thermal energy generated during the propagation of EMWs in absorbers is worth using to enhance the efficiency of energy conversion or improve the thermal management of the absorbers.
- There is a clear need to understand the technical barriers in 1D materials synthesis and preparation, including the growth mechanism of nanostructures, controllable morphology, and issues in scale-up synthesis processes.
- Multifunctional absorbing materials need to be developed to meet diverse requirements in practical applications, such as flame retardancy, waterproof self-cleaning, and oxidation resistance.
- Developing functional unit sequence models of materials will help reveal the construction mechanism of stealth materials and conduct research on intrinsic laws.

## References

[1] Wen C, Li X, Zhang R et al. High-density anisotropy magnetism enhanced microwave absorption performance in Ti_3_C_2_T_x_ MXene@Ni microspheres. ACS Nano 2022; 16: 1150–9. 10.1021/acsnano.1c08957 34957827

[2] Huang M, Wang L, Pei K et al. Multidimension-controllable synthesis of MOF-derived Co@N-doped carbon composite with magnetic-dielectric synergy toward strong microwave absorption. Small 2020; 16: 2000158. 10.1002/smll.202000158 32182407

[3] Iqbal A, Shahzad F, Hantanasirisakul K et al. Anomalous absorption of electromagnetic waves by 2D transition metal carbonitride Ti_3_CNT_x_ (MXene). Science 2020; 369: 446–50. 10.1126/science.aba7977 32703878

[4] Wang L, Li X, Li Q et al. Oriented polarization tuning broadband absorption from flexible hierarchical ZnO arrays vertically supported on carbon cloth. Small 2019; 15: 1900900. 10.1002/smll.201900900 30957426

[5] Liu J, Che R, Chen H et al. Microwave absorption enhancement of multifunctional composite microspheres with spinel Fe_3_O_4_ cores and anatase TiO_2_ shells. Small 2012; 8: 1214–21. 10.1002/smll.201102245 22331748

[6] Zhi C, Zhang S, Wu H et al. Perovskite nanocrystals induced core–shell inorganic–organic nanofibers for efficient energy harvesting and self-powered monitoring. ACS Nano 2024; 18: 9365–77. 10.1021/acsnano.3c09935 38517349

[7] Yang Y, Cheng J, Pan F et al. Phragmites-derived magnetic carbon fiber with hollow assembly architecture toward full-covered effective bandwidth at ku band. Carbon 2023; 213: 118228. 10.1016/j.carbon.2023.118228

[8] Wang L, Li X, Shi X et al. Recent progress of microwave absorption microspheres by magnetic-dielectric synergy. Nanoscale 2021; 13: 2136–56. 10.1039/D0NR06267G 33471004

[9] Zhu B, Cui Y, Lv D et al. Synthesis and electromagnetic wave absorption properties of peanut shell-like SiC fibers. Mater Lett 2020; 263: 127288. 10.1016/j.matlet.2019.127288

[10] Ding C, Shao C, Wu S et al. A review of 1D carbon-based materials assembly design for lightweight microwave absorption. Carbon 2023; 213: 118279. 10.1016/j.carbon.2023.118279

[11] Wu Z, Cheng HW, Jin C et al. Dimensional design and core–shell engineering of nanomaterials for electromagnetic wave absorption. Adv Mater 2022; 34: 2107538. 10.1002/adma.202107538 34755916

[12] Liu L, Zhou K, He P et al. Synthesis and microwave absorption properties of carbon coil–carbon fiber hybrid materials. Mater Lett 2013; 110: 76–9. 10.1016/j.matlet.2013.07.131

[13] Lin CK, Chiou YJ, Tsou SJ et al. One pot self-assembling Fe@PANI core–shell nanowires for radar absorption application. Nanomaterials 2023; 13: 1100. 10.3390/nano13061100 36985994 PMC10052763

[14] Wen H, Jin H, Pan J et al. Hollow FeNi/NiFe_2_O_4_-codoped carbon composite nanorods for electromagnetic wave absorption. ACS Appl Nano Mater 2022; 5: 3406–14. 10.1021/acsanm.1c03972

[15] Liu Q, Cao Q, Bi H et al. CoNi@SiO_2_@TiO_2_ and CoNi@air@TiO_2_ microspheres with strong wideband microwave absorption. Adv Mater 2016; 28: 486–90. 10.1002/adma.201503149 26588359

[16] Jin H, Wen H, Hong Q et al. NiCo@NPC@CF nanocomposites derived from NiCo-MOF/cotton for high-performance electromagnetic wave absorption. J Mater Chem C 2022; 10: 8310–20. 10.1039/D2TC00965J

[17] Kuang J, Xiao T, Hou X et al. Microwave synthesis of worm-like SiC nanowires for thin electromagnetic wave absorbing materials. Ceram Int 2019; 45: 11660–7. 10.1016/j.ceramint.2019.03.040

[18] Xiao D, Chen W, Sun L et al. A flexible and ultra-wideband terahertz wave absorber based on pyramid-shaped carbon nanotube array via femtosecond-laser microprocessing and two-step transfer technique. Adv Mater Interfaces 2022; 9: 2102414. 10.1002/admi.202102414

[19] Lim D, Park J, Lee J et al. Broadband mechanical metamaterial absorber enabled by fused filament fabrication 3D printing. Addit Manuf 2022; 55: 102856. 10.1016/j.addma.2022.102856

[20] Huang M, Wang L, Li X et al. Magnetic interacted interaction effect in mxene skeleton: enhanced thermal-generation for electromagnetic interference shielding. Small 2022; 18: 2201587. 10.1002/smll.202201587 35676238

[21] Ding J, Wang L, Zhao Y et al. Boosted interfacial polarization from multishell TiO_2_@Fe_3_O_4_@PPy heterojunction for enhanced microwave absorption. Small 2019; 15: 1902885. 10.1002/smll.201902885 31310052

[22] Zhang X, Li Y, Liu R et al. High-magnetization FeCo nanochains with ultrathin interfacial gaps for broadband electromagnetic wave absorption at gigahertz. ACS Appl Mater Interfaces 2016; 8: 3494–8. 10.1021/acsami.5b12203 26775668

[23] Che RC, Peng LM, Duan XF et al. Microwave absorption enhancement and complex permittivity and permeability of Fe encapsulated within carbon nanotubes. Adv Mater 2004; 16: 401–5. 10.1002/adma.200306460

[24] Zhang Y, Zhang X, Quan B et al. A facile self-template strategy for synthesizing 1D porous Ni@C nanorods towards efficient microwave absorption. Nanotechnology 2017; 28: 115704. 10.1088/1361-6528/aa5d6f 28205507

[25] Wei H, Zhang Z, Hussain G et al. Techniques to enhance magnetic permeability in microwave absorbing materials. Appl Mater Today 2020; 19: 100596. 10.1016/j.apmt.2020.100596

[26] Panina LV, Zagorskiy DL, Shymskaya A et al. 1D nanomaterials in Fe-group metals obtained by synthesis in the pores of polymer templates: correlation of structure, magnetic, and transport properties. Phys Status Solidi A 2022; 219: 2100538. 10.1002/pssa.202100538

[27] Wang J, Huyan Y, Yang Z et al. Tubular carbon nanofibers: synthesis, characterization and applications in microwave absorption. Carbon 2019; 152: 255–66. 10.1016/j.carbon.2019.06.048

[28] Wang X, Lv X, Liu Z et al. Multi-interfacial 1D magnetic ferrite@C fibers for broadband microwave absorption. Mat Today Phys 2023; 35: 101140. 10.1016/j.mtphys.2023.101140

[29] Wen L, Yan Z, Zhu Y et al. Recent progress on the electromagnetic wave absorption of one-dimensional carbon-based nanomaterials. J Mater Res Technol 2023; 26: 2191–218. 10.1016/j.jmrt.2023.08.029

[30] Jiao Z, Ma M, Bi Y et al. A review of carbon-based magnetic microwave-absorbing composites with one-dimensional structure. J Mater Sci 2022; 57: 18243–65. 10.1007/s10853-022-07803-7

[31] Li L, Chen Z, Pan F et al. Electrospinning technology on one dimensional microwave absorbers: fundamentals, current progress, and perspectives. Chem Eng J 2023; 470: 144236. 10.1016/j.cej.2023.144236

[32] Wang Y, Zhu L, Han L et al. Recent progress of one-dimensional nanomaterials for microwave absorption: a review. ACS Appl Nano Mater 2023; 6: 7107–22. 10.1021/acsanm.3c00818

[33] Wang L, Huang M, Yu X et al. Engineering polarization surface of hierarchical ZnO microspheres via spray-annealing strategy for wide-frequency electromagnetic wave absorption. J Mater Sci Technol 2022; 131: 231–9. 10.1016/j.jmst.2022.05.015

[34] Zeng Q, Wang L, Li X et al. Double ligand MOF-derived pomegranate-like Ni@C microspheres as high-performance microwave absorber. Appl Surf Sci 2021; 538: 148051. 10.1016/j.apsusc.2020.148051

[35] Pang L, Xiao P, Li Z et al. Long-range uniform SiC_x_O_y_ beaded carbon fibers for efficient microwave absorption. ACS Appl Mater Interfaces 2023; 15: 30815–25. 10.1021/acsami.3c05029 37335626

[36] He F, Wang Y, Liu J et al. One-dimensional carbon based nanoreactor fabrication by electrospinning for sustainable catalysis. Exploration 2023; 3: 20220164. 10.1002/EXP.20220164 37933386 PMC10624385

[37] Bi Y, Ma M, Jiao Z et al. Enhancing electromagnetic wave absorption performance of one-dimensional C@Co/N-doped C@PPy composite fibers. Carbon 2022; 197: 152–62. 10.1016/j.carbon.2022.05.061

[38] Zheng H, Zhao X, Jiang Q et al. Research progress in the preparation of electromagnetic wave absorbing and corrosion resistant nanofiber materials by electrospinning. J Ind Eng Chem 2024; 138: 34–48. 10.1016/j.jiec.2024.04.010

[39] Pan J, Guo H, Wang M et al. Shape anisotropic Fe_3_O_4_ nanotubes for efficient microwave absorption. Nano Res 2020; 13: 621–9. 10.1007/s12274-020-2656-5

[40] Huang W, Tong Z, Wang R et al. A review on electrospinning nanofibers in the field of microwave absorption. Ceram Int 2020; 46: 26441–53. 10.1016/j.ceramint.2020.07.193

[41] Na K, Jang K, Kim S et al. Fabrication of electrospun Ni_0.5_Zn_0.5_Fe_2_O_4_ nanofibers using polyvinyl pyrrolidone precursors and electromagnetic wave absorption performance improvement. Polym 2021; 13: 4247. 10.3390/polym13234247 PMC8659655 34883751

[42] Huang X, Zhang J, Lai M et al. Preparation and microwave absorption mechanisms of the NiZn ferrite nanofibers. J Alloys Compd 2015; 627: 367–73. 10.1016/j.jallcom.2014.11.235

[43] Yang B, Fang J, Xu C et al. One-dimensional magnetic FeCoNi alloy toward low-frequency electromagnetic wave absorption. Nano-Micro Lett 2022; 14: 170. 10.1007/s40820-022-00920-7 PMC9392832 35987921

[44] Li M, Song X, Xue J et al. Construction of hollow carbon nanofibers with graphene nanorods as nano-antennas for lower-frequency microwave absorption. ACS Appl Mater Interfaces 2023; 15: 31720–8. 10.1021/acsami.3c04839 37356111

[45] Jin C, Wu Z, Yang C et al. Impedance amelioration of coaxial-electrospun TiO_2_@Fe/C@TiO_2_ vesicular carbon microtubes with dielectric-magnetic synergy toward highly efficient microwave absorption. Chem Eng J 2022; 433: 133640. 10.1016/j.cej.2021.133640

[46] Cui Y, Xu K, Zhu B et al. Synthesis of niobium nitride porous nanofibers with excellent microwave absorption properties via reduction nitridation of electrospinning precursor nanofibers with ammonia gas. J Alloys Compd 2022; 907: 164453. 10.1016/j.jallcom.2022.164453

[47] Wu H, Qin M, Zhang L. NiCo_2_O_4_ constructed by different dimensions of building blocks with superior electromagnetic wave absorption performance. Compos Part B-Eng 2020; 182: 107620. 10.1016/j.compositesb.2019.107620

[48] Ma M, Li W, Tong Z et al. 1D flower-like Fe_3_O_4_@SiO_2_@MnO_2_ nanochains inducing RGO self-assembly into aerogels for high-efficient microwave absorption. Mater Des 2020; 188: 108462. 10.1016/j.matdes.2019.108462

[49] Chen Z, Tian K, Zhang C et al. In-situ hydrothermal synthesis of NiCo alloy particles@hydrophilic carbon cloth to construct corncob-like heterostructure for high-performance electromagnetic wave absorbers. J Colloid Interface Sci 2022; 616: 823–33. 10.1016/j.jcis.2022.02.086 35248969

[50] Shen J, Yao Y, Liu Y et al. Tunable hierarchical Fe nanowires with a facile template-free approach for enhanced microwave absorption performance. J Mater Chem C 2016; 4: 7614–21. 10.1039/C6TC01912A

[51] Chen C, Dong H, Wang J et al. A general way to fabricate chain-like ferrite with ultralow conductive percolation threshold and wideband absorbing ability. Nanomaterials 2022; 12: 1603. 10.3390/nano12091603 35564318 PMC9104183

[52] Zhao X, Huang Y, Liu X et al. Magnetic nanorods/carbon fibers heterostructures coated with flower-like MoS_2_ layers for superior microwave absorption. Carbon 2023; 213: 118265. 10.1016/j.carbon.2023.118265

[53] Qiu J, Liang Y, Xiang Y et al. Confined in-situ encapsulation of Co/C composites with increased heterogeneous interface polarization for enhanced electromagnetic performance. Small 2024; 20: 202308270. 10.1002/smll.202308270 37948414

[54] Zhai N, Luo J, Shu P et al. 1D/2D CoTe_2_@MoS_2_ composites constructed by CoTe_2_ nanorods and MoS_2_ nanosheets for efficient electromagnetic wave absorption. Nano Res 2023; 16: 10698–706. 10.1007/s12274-023-5777-9

[55] Zhan Y, Xia L, Yang H et al. Tunable electromagnetic wave absorbing properties of carbon nanotubes/carbon fiber composites synthesized directly and rapidly via an innovative induction heating technique. Carbon 2021; 175: 101–11. 10.1016/j.carbon.2020.12.080

[56] Yan B, Yue J, Fan B et al. Constructing La-doped CoFe_2_O_4_/CNTs hybrids with urchin structure for enhanced microwave absorption performance. J Mater Res 2023; 38: 2908–18. 10.1557/s43578-023-01035-4

[57] Mohd Idris F, Amin Matori K, Ismail I et al. Materials’ characterization and properties of multiwalled carbon nanotubes from industrial waste as electromagnetic wave absorber. J Nanopart Res 2022; 24: 244. 10.1007/s11051-022-05625-x

[58] Wang Z, Liu J, Hao H et al. Microwave absorption enhancement by SiC nanowire aerogels through heat treatment-based oxidation modulation. Carbon 2024; 217: 118622. 10.1016/j.carbon.2023.118622

[59] He P, Hou Z, Cao W et al. Rutile TiO_2_ nanorod with anomalous resonance for charge storage and frequency selective absorption. Ceram Int 2021; 47: 2016–21. 10.1016/j.ceramint.2020.09.033

[60] Qin M, Liang H, Zhao X et al. Filter paper templated one-dimensional NiO/NiCo_2_O_4_ microrod with wideband electromagnetic wave absorption capacity. J Colloid Interface Sci 2020; 566: 347–56. 10.1016/j.jcis.2020.01.114 32018175

[61] Liang L, Han G, Li Y et al. Promising Ti_3_C_2_T_x_ MXene/Ni chain hybrid with excellent electromagnetic wave absorption and shielding capacity. ACS Appl Matter Interfaces 2019; 11: 25399–409. 10.1021/acsami.9b07294 31259512

[62] Dai S, Quan B, Zhang B et al. Constructing multi-interface Mo_2_C/Co@C nano-rods for a microwave response based on a double attenuation mechanism. Dalton Trans 2019; 47: 14767–73. 10.1039/C8DT03282C 30294732

[63] Zhang F, Zhang L, Fan Y et al. Fabrication of multiple core-shell structures MnO@HsGDY@NC@HsGDY hybrid nanofibers for enhanced microwave absorption. Carbon 2024; 216: 118588. 10.1016/j.carbon.2023.118588

[64] Wen L, Guan L, Zhang J et al. Amorphous carbon regulates defects to achieve TaC nanowires with enhanced microwave absorption. ACS Appl Nano Mater 2024; 7: 1357–68. 10.1021/acsanm.3c05486

[65] Gholipur R, Taha NA, Afrouzeh K. Synthesis and identification of structural, optical, electrical, magnetic, photocatalytic, and electrochemical properties of novel Au/MoS_2_@(NiFe_2_O_4_)_x_ nanocomposites. Sensors Actuat A-Phys 2023; 362: 114645. 10.1016/j.sna.2023.114645

[66] Li Y, Wang J, Li H et al. Effect of conductive PANI vs. insulative PS shell coated Ni nanochains on electromagnetic wave absorption. J Alloys Compd 2020; 821: 153531. 10.1016/j.jallcom.2019.153531

[67] Tahalyani J, Akhtar MJ, Kar KK. Flexible, stretchable, and lightweight hierarchical carbon-nanotube-decorated carbon fiber structures for microwave absorption. ACS Appl Nano Mater 2023; 6: 11888–901. 10.1021/acsanm.3c01746

[68] Wu Z, Pei K, Xing L et al. Enhanced microwave absorption performance from magnetic coupling of magnetic nanoparticles suspended within hierarchically tubular composite. Adv Funct Mater 2019; 29: 1901448. 10.1002/adfm.201901448

[69] Yang N, Zeng J, Xue J et al. Strong absorption and wide-frequency microwave absorption properties of the nanostructure zinc oxide/zinc/carbon fiber multilayer composites. J Alloys Compd 2018; 735: 2212–8. 10.1016/j.jallcom.2017.11.380

[70] Javid M, Zhou Y, Zhou T et al. In-situ fabrication of Fe@ZrO_2_ nanochains for the heat-resistant electromagnetic wave absorber. Mater Lett 2019; 242: 199–202. 10.1016/j.matlet.2019.01.053

[71] Qiao J, Zhang X, Xu D et al. Design and synthesis of TiO_2_/Co/carbon nanofibers with tunable and efficient electromagnetic absorption. Chem Eng J 2020; 380: 122591. 10.1016/j.cej.2019.122591

[72] Li F, Zhang H. Synthesis of hollow sphere and 1D structural materials by sol-gel process. Materials 2017; 10: 995. 10.3390/ma10090995 28841188 PMC5615650

[73] Liao Z, Ma M, Tong Z et al. Fabrication of one-dimensional ZnFe_2_O_4_@carbon@MoS_2_/FeS_2_ composites as electromagnetic wave absorber. J Colloid Interface Sci 2021; 600: 90–8. 10.1016/j.jcis.2021.04.142 34004433

[74] Zhao L, Guo Y, Xie Y et al. Construction of SiCNWS@NiCo_2_O_4_@PANI 1D hierarchical nanocomposites toward high-efficiency microwave absorption. Appl Surf Sci 2022; 592: 153324. 10.1016/j.apsusc.2022.153324

[75] Li J, Luo Y, Wang C et al. Confined pyrolysis-driven one-dimensional carbon structure evolution from polyacrylonitrile fiber and its microwave absorption performance. Carbon 2024; 218: 118751. 10.1016/j.carbon.2023.118751

[76] Deng Y, Yang M, Liu Q et al. Biologically inspired nanoporous PAN/PMMA/β-CD carbon fibers for efficient microwave absorption. ACS Appl Nano Mater 2024; 7: 3199–209. 10.1021/acsanm.3c05523

[77] Xiao F, Sun H, Li J et al. Electrospinning preparation and electromagnetic wave absorption properties of SiCN fibers. Ceram Int 2020; 46: 12773–81. 10.1016/j.ceramint.2020.02.046

[78] Tong G, Wu W, Guan J et al. Solution synthesis and novel magnetic properties of ball-chain iron nanofibers. J Mater Res 2011; 26: 2590–8. 10.1557/jmr.2011.307

[79] Sun J, Aslani F, Wei J et al. Electromagnetic absorption of copper fiber oriented composite using 3D printing. Constr Build Mater 2021; 300: 124026. 10.1016/j.conbuildmat.2021.124026

[80] Ma M, Hu J, Han X et al. Design and synthesis of one-dimensional magnetic composites with Co nanoparticles encapsulated in carbon nanofibers for enhanced microwave absorption. J Colloid Interface Sci 2023; 652: 680–91. 10.1016/j.jcis.2023.08.031 37573239

[81] Hu J, Jiao Z, Jiang J et al. Simple fabrication of cobalt–nickel alloy/carbon nanocomposite fibers for tunable microwave absorption. J Colloid Interface Sci 2023; 652: 1825–35. 10.1016/j.jcis.2023.09.009 37683410

[82] Yang G, Wen B, Zhou Z et al. Flexible cobalt nanoparticles/carbon nanofibers with macroporous structures toward superior electromagnetic wave absorption. J Colloid Interface Sci 2023; 636: 194–203. 10.1016/j.jcis.2022.12.158 36630856

[83] Guan G, Li X, Li Y et al. Ultrasmall SnFe_2_O_4_ nanoparticles anchored on N-doped carbon nanofibers for ultralight and high-performance microwave absorption. Phys Chem Chem Phys 2023; 25: 30832–7. 10.1039/D3CP02657D 37962012

[84] Li H, Wu A, Qiu Z et al. Carbonization of Ni@SiC@C nanoparticles reinforced PAN nanofibers for adjustable impedance matching. Chem Eng J 2023; 476: 146582. 10.1016/j.cej.2023.146582

[85] Wang S, Liu Q, Li K et al. Joule-heating-driven synthesis of a honeycomb-like porous carbon nanofiber/high entropy alloy composite as an ultralightweight electromagnetic wave absorber. ACS Nano 2024; 18: 5040–50. 10.1021/acsnano.3c11408 38286018

[86] Zhao B, Yan Z, Du Y et al. High-entropy enhanced microwave attenuation in titanate perovskites. Adv Mater 2023; 35: 2210243. 10.1002/adma.202210243 36606342

[87] Qiu Z, Liu X, Yang T et al. Synergistic enhancement of electromagnetic wave absorption and corrosion resistance properties of high entropy alloy through lattice distortion engineering. Adv Funct Mater 2024; 34: 2400220. 10.1002/adfm.202400220

[88] Chen J, Sun C, Han Z et al. Rapidly synthesizing magneto-thermal adjustable high entropy alloy nanoparticles on carbon fiber surface for enhancing electromagnetic wave absorption, thermal conductivity and interface compatibility of composites. Chem Eng J 2024; 498: 155351. 10.1016/j.cej.2024.155351

[89] Wang H, Xiao X, An Q et al. Low-frequency evolution mechanism of customized HEAs-based electromagnetic response modes manipulated by carbothermal shock. Small 2024; 20: 2309773. 10.1002/smll.202309773 38461545

[90] Zhang X, Gong M, Dai Y et al. Construction of one-dimensional MoO_2_/NC heteronanowires for microwave absorption. Rsc Adv 2022; 12: 5157–63. 10.1039/D1RA09074G 35425555 PMC8981422

[91] Dou Q, Zhang X, Wang Y et al. Structure control and growth mechanism of beaded SiC nanowires with microwave absorption properties. Mat Sci Eng B 2023; 296: 116657. 10.1016/j.mseb.2023.116657

[92] Liu Y, Jia Z, Zhou J et al. Multi-hierarchy heterostructure assembling on MnO_2_ nanowires for optimized electromagnetic response. Mat Today Phys 2022; 28: 100845. 10.1016/j.mtphys.2022.100845

[93] Yang P, Cai R, Ruan H et al. In situ synthesis of heterostructured Fe–Ni nanowires with tunable electromagnetic wave absorption capabilities. ACS Appl Nano Mater 2023; 6: 14322–31. 10.1021/acsanm.3c02321

[94] Qian Y, Meng X, Liu H et al. Magnetic field-induced synthesis of one-dimensional nickel nanowires for enhanced microwave absorption. Adv Mater Interfaces 2023; 10: 2201604. 10.1002/admi.202201604

[95] Arief I, Biswas S, Bose S. Tuning the shape anisotropy and electromagnetic screening ability of ultrahigh magnetic polymer and surfactant-capped FeCo nanorods and nanocubes in soft conducting composites. ACS Appl Matter Interfaces 2016; 8: 26285–97. 10.1021/acsami.6b07464 27602950

[96] Wu W, Liu Y, Zhou Q et al. Microwave absorbing properties of FeB/B_4_C nanowire composite. Ceram Int 2020; 46: 4020–3. 10.1016/j.ceramint.2019.10.052

[97] Yang P, Ye W, Ruan H et al. Core–shell structured silica-coated iron nanowires composites for enhanced electromagnetic wave absorption properties. Int J Mol Sci 2023; 24: 8620. 10.3390/ijms24108620 37239958 PMC10217952

[98] Pan F, Ning M, Li Z et al. Sequential architecture induced strange dielectric-magnetic behaviors in ferromagnetic microwave absorber. Adv Funct Mater 2023; 33: 2300374. 10.1002/adfm.202300374

[99] Zhao B, Li Y, Zeng Q et al. Galvanic replacement reaction involving core–shell magnetic chains and orientation-tunable microwave absorption properties. Small 2020; 16: 2003502. 10.1002/smll.202003502 32893495

[100] Dai B, Ma Y, Feng S et al. Fabrication of one-dimensional M (Co, Ni)@polyaniline nanochains with adjustable thickness for excellent microwave absorption properties. J Colloid Interface Sci 2022; 627: 113–25. 10.1016/j.jcis.2022.06.137 35842962

[101] Pan F, Yu L, Xiang Z et al. Improved synergistic effect for achieving ultrathin microwave absorber of 1D Co nanochains/2D carbide MXene nanocomposite. Carbon 2021; 172: 506–15. 10.1016/j.carbon.2020.10.039

[102] Li C, Li D, Zhang S et al. Interface engineering of titanium nitride nanotube composites for excellent microwave absorption at elevated temperature. Nano-Micro Lett 2024; 16: 168. 10.1007/s40820-024-01381-w PMC10994892 38573346

[103] Xu S, Liao P, Zhu J et al. Co nanoparticles embedded in N-doped carbon nanotubes for broadband microwave absorption. ACS Appl Nano Mater 2024; 7: 8671–84. 10.1021/acsanm.3c06210

[104] Li C, Zhang S, Wang X et al. Efficient and thin microwave absorption materials fabricated by polyzwitterion wrapped carbon nanotube. Appl Surf Sci 2022; 600: 154060. 10.1016/j.apsusc.2022.154060

[105] Hu J, Zhao T, Peng X et al. Growth of coiled amorphous carbon nanotube array forest and its electromagnetic wave absorbing properties. Compos Part B-Eng 2018; 134: 91–7. 10.1016/j.compositesb.2017.09.071

[106] Cheng J, Yuan W, Zhang A et al. Porous CoNi nanoalloy@N-doped carbon nanotube composite clusters with ultra-strong microwave absorption at a low filler loading. J Mater Chem C 2020; 8: 13712–22. 10.1039/D0TC03377D

[107] Li C, Zhang S, Yi J et al. Facile-prepared imidazole-based ionic liquid/CNT composites with high-electromagnetic wave absorption performance. J Mater Sci-mater El 2023; 34: 1306. 10.1007/s10854-023-10637-x

[108] Hu Q, Yang R, Yang S et al. Metal–organic framework-derived core–shell nanospheres anchored on Fe-filled carbon nanotube sponge for strong wideband microwave absorption. ACS Appl Matter Interfaces 2022; 14: 10577–87. 10.1021/acsami.1c25019 35188369

[109] Wang J, Jia Z, Liu X et al. Construction of 1D heterostructure NiCo@C/ZnO nanorod with enhanced microwave absorption. Nano-Micro Lett 2021; 13: 175. 10.1007/s40820-021-00704-5 PMC8368508 34398334

[110] Wang W, Nan K, Zheng H et al. Heterostructure design of one-dimensional ZnO@CoNi/C multilayered nanorods for high-efficiency microwave absorption. J Colloid Interface Sci 2024; 657: 491–501. 10.1016/j.jcis.2023.11.186 38070335

[111] Sun M, Wang D, Xiong Z et al. Multi-dimensional Ni@C-CoNi composites with strong magnetic interaction toward superior microwave absorption. J Mater Sci Technol 2022; 130: 176–83. 10.1016/j.jmst.2022.05.016

[112] Wu N, Xu D, Wang Z et al. Achieving superior electromagnetic wave absorbers through the novel metal-organic frameworks derived magnetic porous carbon nanorods. Carbon 2019; 145: 433–44. 10.1016/j.carbon.2019.01.028

[113] Zhao B, Wu N, Yao S et al. Molybdenum carbide/cobalt composite nanorods via a “MOFs plus MOFs” strategy for high-efficiency microwave absorption. ACS Appl Nano Mater 2022; 5: 18697–707. 10.1021/acsanm.2c04460

[114] Zhang R, Guo D, Liu Q et al. Engineering core–shell heterocomposites of integrated 0D ZIF-8 (Zn) attached on 1D MIL-68 (In) nanorods for high performance electromagnetic wave absorption. Appl Surf Sci 2023; 621: 156898. 10.1016/j.apsusc.2023.156898

